# p53 activates circASCC3 to repress R-loops and enhance resistance to chemotherapy

**DOI:** 10.1073/pnas.2415869122

**Published:** 2025-03-11

**Authors:** Mingming Cao, Yu Gan, Yingdan Huang, Jing Tong, Chen Xiong, Yajie Chen, Bing Chen, Ruixuan Huang, Bangxiang Xie, Jun Deng, Shenglin Huang, Xianghuo He, Qian Hao, Xiang Zhou

**Affiliations:** ^a^Fudan University Shanghai Cancer Center and Institutes of Biomedical Sciences, Fudan University, Shanghai 200032, China; ^b^Department of Oncology, Shanghai Medical College, Fudan University, Shanghai 200032, China; ^c^Department of Radiation Oncology, Fudan University Shanghai Cancer Center, Shanghai 200032, China; ^d^Department of Oncology, The First Affiliated Hospital, Jiangxi Medical College, Nanchang University, Nanchang 330006, Jiangxi, China; ^e^Jiangxi Key Laboratory for Individual Cancer Therapy, Nanchang 330006, Jiangxi, China; ^f^Beijing Institute of Hepatology, Beijing Youan Hospital, Capital Medical University, Beijing 100069, China; ^g^Beijing Engineering Research Center for Precision Medicine and Transformation of Hepatitis and Liver Cancer, Beijing 100069, China; ^h^Key Laboratory of Breast Cancer in Shanghai, Fudan University Shanghai Cancer Center, Fudan University, Shanghai 200032, China; ^i^Shanghai Key Laboratory of Medical Epigenetics, International Co-laboratory of Medical Epigenetics and Metabolism (Ministry of Science and Technology), Institutes of Biomedical Sciences, Fudan University, Shanghai 200032, China

**Keywords:** p53, circular RNA, DDX5, R-loop, chemoresistance

## Abstract

The tumor suppressor p53 can trigger tumor resistance to chemotherapy by facilitating DNA damage repair and maintaining genomic integrity. Here, we report that a p53-induced circular RNA circASCC3 promotes chemotherapeutic resistance by resolving R-loops. Our results reveal that p53 directly activates the transcription of *ASCC3*, the host gene of circASCC3. In addition, the RNA-binding protein SFPQ is identified to inhibit the formation of circASCC3 by associating with its flanking regions. Importantly, p53 facilitates the formation of circASCC3 by repressing the expression of SFPQ. CircASCC3 has a marginal effect on the survival and growth of cancer cells under normal growing conditions but surprisingly boosts their survival and growth in response to DNA damage stress. Mechanistic analysis reveals that circASCC3 binds to the DEAD-box RNA helicase DDX5 to inhibit its proteasomal degradation. This results in the prevention of R-loop accumulation due to DNA damage, thereby conferring tumor resistance to chemotherapy. Together, our study uncovers that p53 activates circASCC3 to promote R-loop resolution, which maintains genomic stability and potentially contributes to chemoresistance.

## Significance

The tumor suppressor p53 facilitates the repair of DNA damage, thereby preventing malignant transformation or inducing resistance to chemotherapy in cancer. This study reveals that p53 activates the expression of the circular RNA circASCC3, protecting cancer cells from apoptosis upon DNA damage. Mechanistically, circASCC3 interacts with and enhances the stability of DDX5 protein, a DEAD-box RNA helicase that can prevent R-loop accumulation. This study uncovers a key role of the p53–circASCC3 axis in maintaining genomic stability.

Genomic instability is causative for malignant transformation and cancer progression. Defects in the DNA damage repair system result in chromosomal alterations, gene mutations, and tumorigenesis (1–3). However, deficiencies in DNA repair genes can also drive apoptosis in cancer cells exposed to chemotherapy, because a compromised repair system fails to detect or resolve damaged DNA (4). For example, while BRCA1/2 mutation or homologous recombination deficiency increases cancer predisposition, such deficiencies can enhance tumor sensitivity to genotoxic therapeutics (5). Therefore, a robust DNA repair system can prevent cancer initiation, while also protecting cancer cells from genotoxic insults and consequent apoptosis.

The tumor suppressor p53 prevents tumorigenesis by maintaining genomic integrity and is thus regarded as the “guardian of the genome” (6). Upon moderate genotoxic stresses, p53 induces cell cycle arrest and promotes the repair of damaged DNA (7, 8). In this means, p53 maintains genomic stability and prevents cells from malignant transformation. In contrast, germline mutation of *TP53* gene leads to increased genomic instability and a familial cancer-prone disease called Li–Fraumeni syndrome (9, 10). However, recent evidence has revealed that p53 can also confer drug resistance in cancer by facilitating DNA repair through various mechanisms. For instance, p53 induces the transcription of nucleotide excision repair genes, *XPC* and *DDB2*, to promote melanoma resistance to chemotherapeutics (11). Another p53 target gene *MGMT*, encoding O-6-methylguanine-DNA methyltransferase, facilitates the repair of DNA alkylation damage, resulting in tumor resistance to alkylating agents (12). In addition, p53 can prevent aberrant chromosomal alterations by activating a long noncoding RNA (lncRNA) GUARDIN (13). p53 promotes the resistance to genotoxic stress, particularly when it is not completely activated (7, 14). It has been reported that RMRP limits the full activation of p53, potentially leading to cell cycle arrest and DNA damage repair, which ultimately triggers tumor resistance to PARP inhibitors (15, 16).

R-loop is an RNA/DNA hybrid structure which is formed during transcription when an RNA strand invades double-stranded DNA. Accumulation of R-loops due to DNA damage, oncogenic activation, or the dysfunction of the R-loop removing machinery leads to transcription-replication conflicts, replication stress, and compromised double-strand break repair (17, 18). Recently, it was reported that p53 prevented R-loop-associated genomic instability by restricting aberrant satellite transcription (19). In addition, an elevated level of R-loops due to the inactivation of p53 by the E6 viral oncoprotein was found to be necessary for HPV replication and pathogenesis (20). These studies suggest a crucial yet understudied role of p53 in the regulation of unscheduled R-loop formation. In our study as presented here, we identified that a p53-inducible circular RNA circASCC3 increased the survival and growth of cancer cells upon DNA damage stress. Mechanistically, circASCC3 interacted with and stabilizes the DEAD-box RNA helicase DDX5, leading to the resolution of R-loops. Our study uncovers an essential role for circASCC3 in p53-mediated R-loop resolution and genomic stability.

## Results

### Identification of circASCC3 as a p53-Induced Circular RNA.

**Fig. 1.**
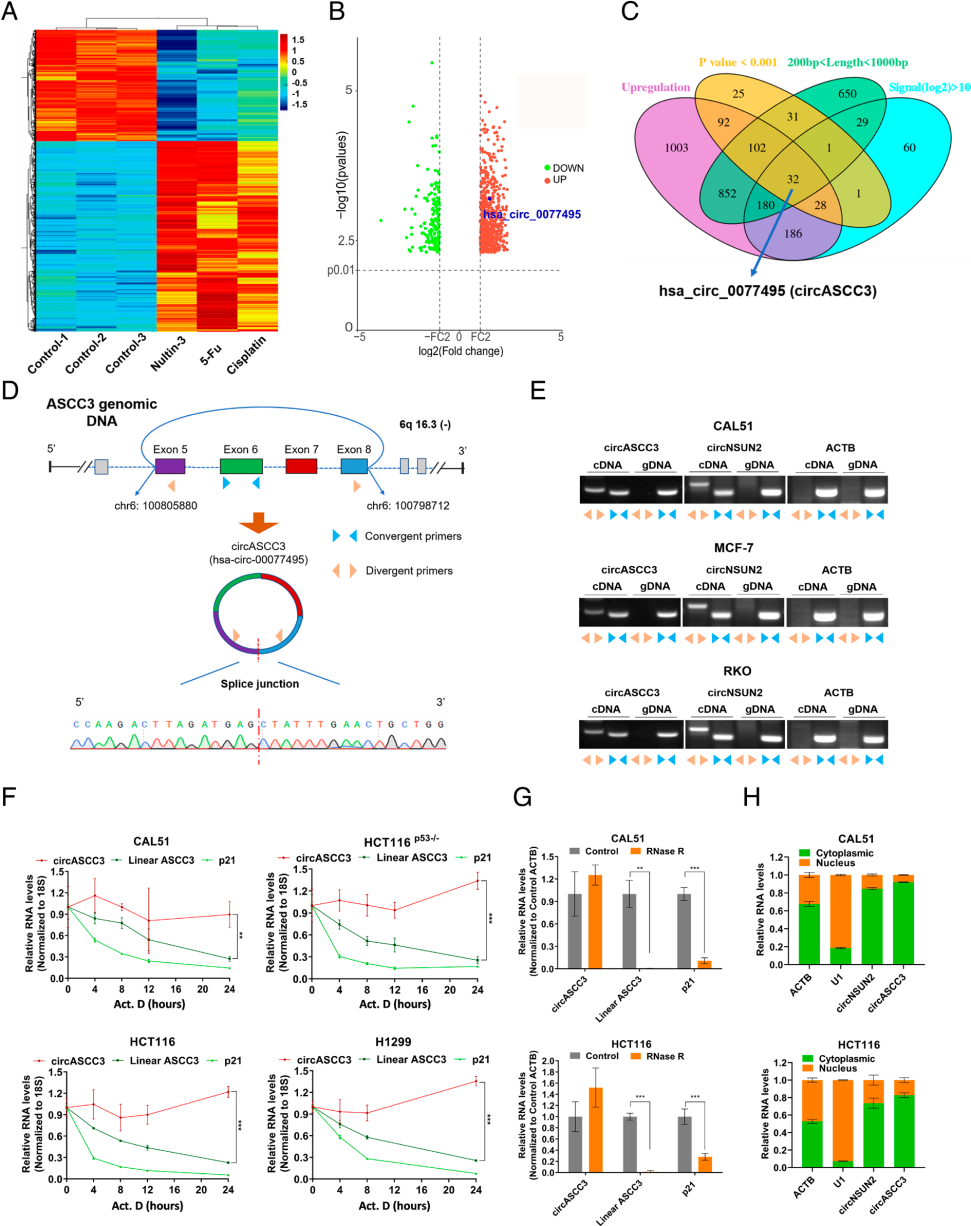
CircASCC3 is a circular RNA responsive to DNA damage. (*A*) Heatmap of differentially expressed circRNAs in cancer cells when treated with DMSO, Nutlin-3 (10 μM), Cisplatin (10 μM), or 5-FU (20 μM). (*B*) The volcano plot of circRNAs that are differentially expressed upon the treatments with all three agents (Nutlin-3, Cisplatin, and 5-FU). (*C*) Venn diagram of circRNAs based on the indicated criteria. (*D*) The genomic locus of circASCC3 and sequencing analysis of the head-to-tail splicing junction in circASCC3. Blue arrows represent convergent primers, and yellow arrows represent divergent primers. (*E*) CircASCC3 is amplified from cDNAs using divergent primers, rather than from gDNA. circNSUN2 served as a positive control and ACTB as a negative control. (*F*) CircASCC3 is more stable than ASCC3 and p21 mRNAs. (*G*) CircASCC3 is resistant to RNase R digestion compared to ASCC3 and p21 mRNAs. (*H*) CircASCC3 is mainly localized to the cytoplasm. ACTB, U1, and circNSUN2 served as references for comparison. **P < 0.01, ***P < 0.001.

To identify circular RNAs that are responsive to DNA damage and p53 activation, we conducted a whole-transcriptome microarray analysis of CAL51 cells that were treated with the chemotherapeutic agents, Nutlin-3, 5-fluorouracil (5-FU), or Cisplatin (Fig. 1*A*). The results were reliable, because numerous known p53-inducible protein-coding genes were upregulated upon the treatments (*SI Appendix*, Fig. S1*A*). Through a combined analysis of the fold changes, *P* values, and the length and expression of circular RNAs (Fig. 1 *B* and *C* and *SI Appendix*, Fig. S1 *B–D*), we identified that the baseline expression level of the circular RNA hsa_circ_0077495 was relatively high (as determined by microarray data and cycle threshold (Ct) values of RT-qPCR) and could be consistently upregulated by Nutlin-3 and DNA damage-inducing agents. This circular RNA was named as circASCC3, because it consists of the exons 5 to 8 of the host gene *ASCC3*, which was validated by Sanger sequencing (Fig. 1*D*). The circular structure of circASCC3 was further confirmed by PCR analysis using both convergent and divergent primers as described (Fig. 1*D*). Our results showed that the PCR products from the junction site could be amplified from cDNA, but not from genomic DNA, using the divergent primers, indicating the presence of a backsplicing event at this site (Fig. 1*E*). CircNSUN2 served as a reference for comparison (21). In addition, circASCC3 was more stable than the linear transcripts of *ASCC3* and *CDKN1A* (usually known as p21), as it exhibited prolonged half-life in cancer cells when transcription was blocked by Actinomycin D (Fig. 1*F*) and was resistant to RNase R digestion (Fig. 1*G*). Finally, we determined the subcellular localization of circASCC3 and found that this circular RNA was mainly distributed in the cytoplasm of cancer cells (Fig. 1*H* and *SI Appendix*, Fig. S1*E*). These results indicate that circASCC3 is a circular RNA whose expression is elevated in response to DNA damage and p53 activation.

### p53 Transcriptionally Activates circASCC3 Expression.

**Fig. 2.**
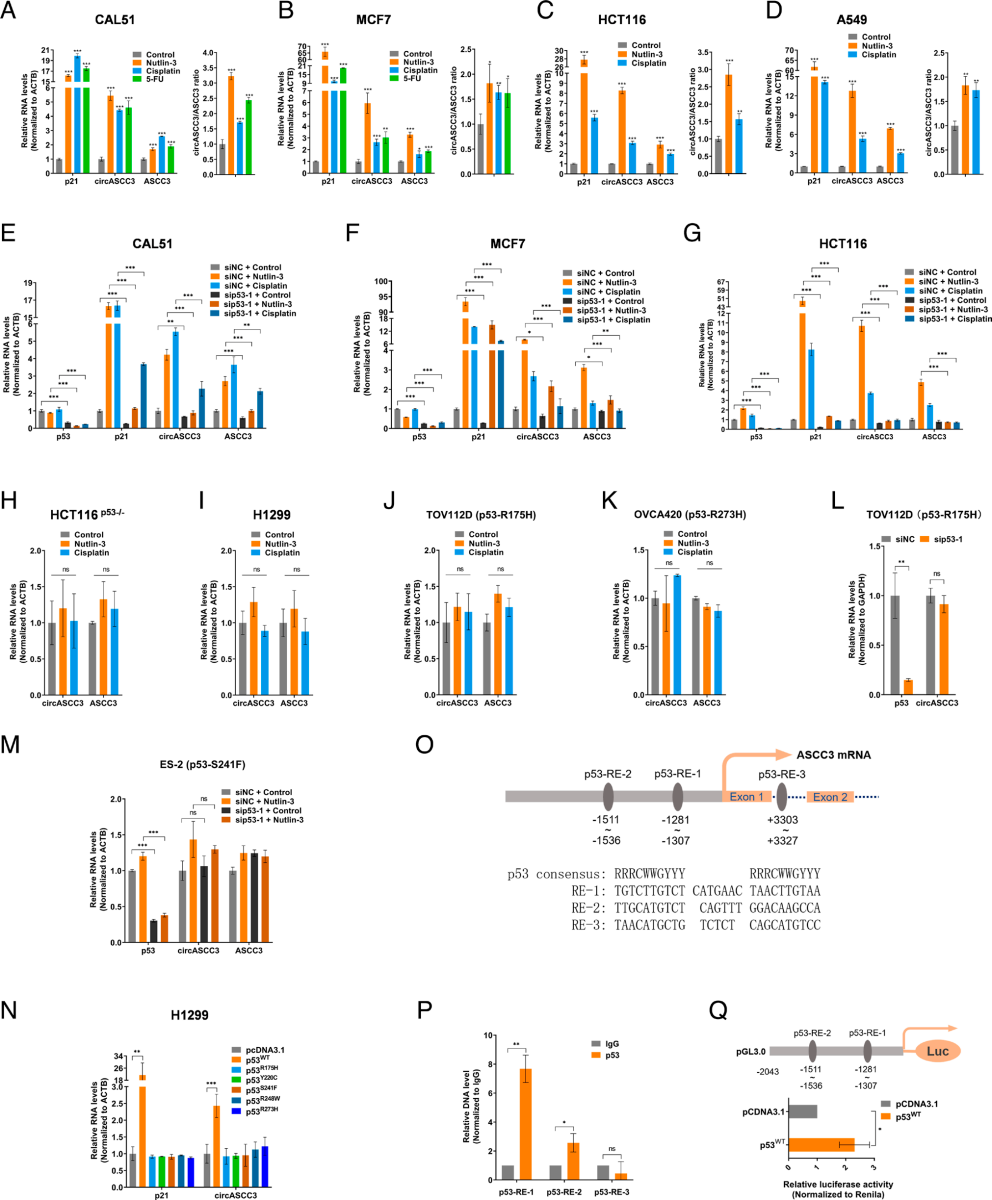
p53 transcriptionally induces the expression of circASCC3. (*A*–*D*) The expression of circASCC3 and ASCC3 mRNA is elevated upon Nutlin-3 or DNA damage-inducing agent treatment. CAL51 (A), MCF7 (*B*), HCT116 (*C*), and A549 (*D*) cells were treated with Nutlin-3 (10 μM), Cisplatin (10 μM), or 5-FU (20 μM) for 48 h, followed by RT-qPCR analysis. Right panels indicate relative abundance of circASCC3 by normalizing to ASCC3 mRNA. (*E*–*G*) Knockdown of p53 abolishes the elevation of both circASCC3 and ASCC3 mRNA levels. (*H*–*K*) The levels of circASCC3 and ASCC3 mRNA are unaffected by Nutlin-3 (10 μM) or Cisplatin (10 μM) treatment in p53-null or -mutated cancer cells. (*L* and *M*) Knockdown of p53 has no impact on the expression of circASCC3 in p53-mutated cancer cells. (*N*) Overexpression of WT p53, but not mutant p53s, upregulates the expression of circASCC3 in H1299 cells. (*O*) Schematic illustration of the potential p53-responsive elements (p53-REs) within the promoter and the first intron of *ASCC3*. (*P*) p53 binds to p53-RE-1 and -2, determined by the ChIP assay. (*Q*) Overexpression of p53 triggers luciferase activity driven by the ASCC3 promoter, determined by the luciferase reporter assay. *P < 0.05, **P < 0.01, ***P< 0.001.

To investigate whether p53 regulates the expression of circASCC3, we performed a set of RT-qPCR analysis following the treatment of cancer cells with p53-inducing agents. These agents markedly induced the expression of circASCC3 and its host gene *ASCC3*, as well as the p53 target gene p21 in a panel of wild-type (WT) p53-harboring cancer cells (Fig. 2 *A–D* and *SI Appendix*, Fig. S2*A*). In contrast, knockdown of p53 using two independent siRNAs significantly reduced the expression of circASCC3 and *ASCC3* in cancer cells when treated with Cisplatin or Nutlin-3 (Fig. 2 *E*–*G* and *SI Appendix*, Fig. S2 *B–D*). We also tested whether WT p53 was required for this regulation by employing cancer cells without p53 or expressing a mutant p53. Treatment of these cells with Cisplatin or Nutlin-3 did not affect the expression of circASCC3 and *ASCC3* (Fig. 2 *H*–*K*). In addition, knockdown of mutant p53 had no significant impact on the expression of circASCC3 and *ASCC3* (Fig. 2 *L* and *M*). Consistently, overexpression of WT p53, but not a number of p53 mutants, dramatically induced the expression of circASCC3 (Fig. 2*N*). These results suggested that the increase in circASCC3 expression might be associated with the transcriptional activity of WT p53. To test this hypothesis, we searched the promoter sequence of *ASCC3* for potential p53-responsive elements (p53-REs) using the p53MH algorithm (22). Three possible p53-REs were identified at the positions, −1,511 to −1,536, −1,281 to −1,307, and +3,303 to +3,327 (Fig. 2*O*). The ChIP assay was performed to show that p53 strongly bound to p53-RE-1 and moderately to p53-RE-2, but not bound to p53-RE-3 (Fig. 2*P*). Also, overexpression of p53 significantly induced luciferase activity driven by the *ASCC3* promoter fragment encompassing p53-RE-1 and -2 (Fig. 2*Q*). Together, these results demonstrate that p53 induces the expression of circASCC3 by transcriptionally activating its host gene *ASCC3*.

### The RNA-Binding Protein SFPQ Is Involved in p53-Mediated Upregulation of circASCC3.

**Fig. 3.**
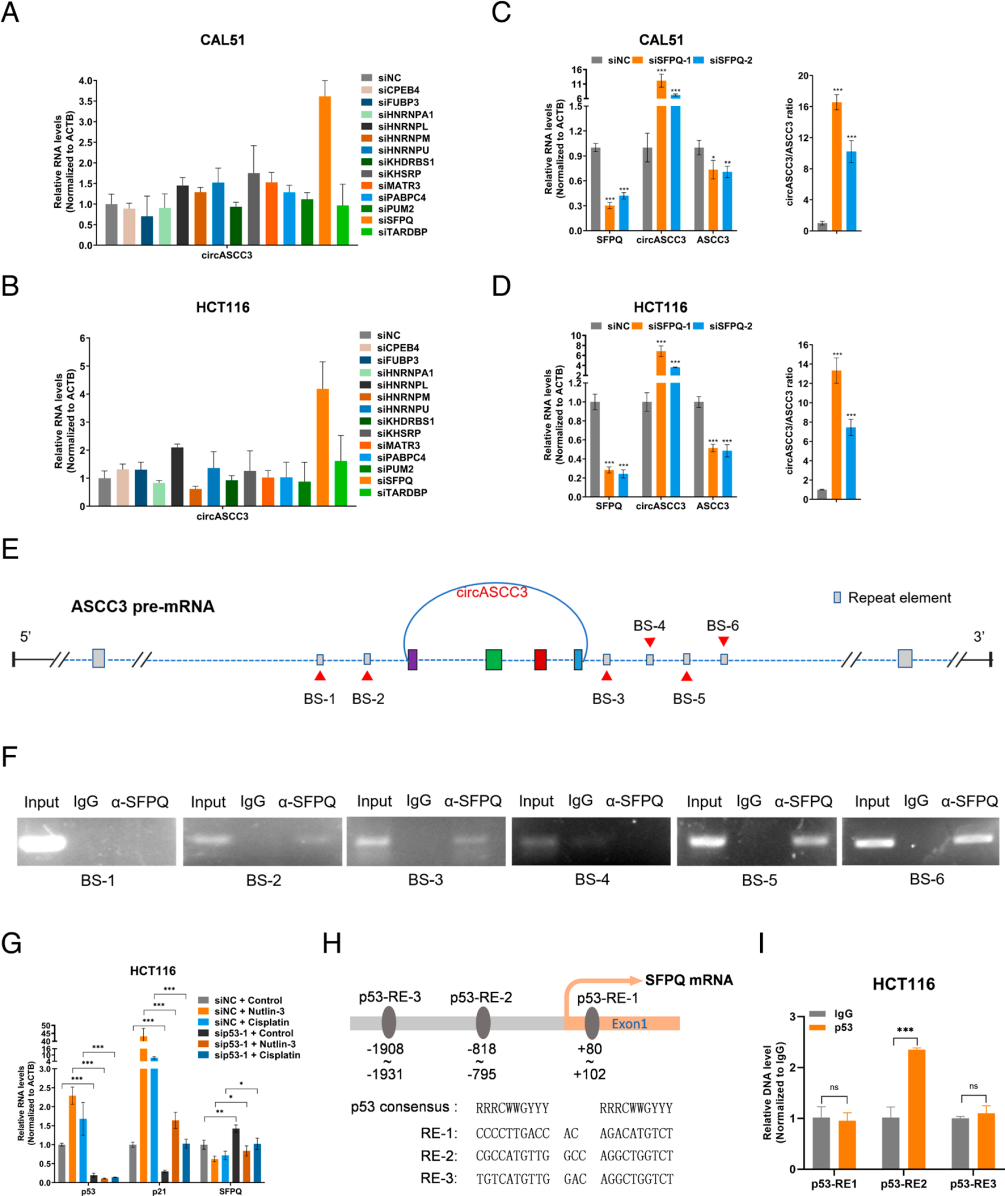
SFPQ is involved in p53-mediated upregulation of circASCC3. (*A* and *B*) The levels of circASCC3 were measured after knocking down the expression of a panel of RBPs. (*C* and *D*) Knockdown of SFPQ elevates the levels of circASCC3 but reduces ASCC3 mRNA expression. Right panels indicate relative abundance of circASCC3 by normalizing to ASCC3 mRNA. (*E*) Schematic illustration of the repeat elements and potential SFPQ-binding sites within the repeat elements near the flanking regions of circASCC3. (*F*) SFPQ associates with the repeat elements, as determined by the RIP assay. (*G*) Knockdown of p53 elevates the levels of SFPQ mRNA. (*H*) Schematic illustration of the potential p53-REs within the promoter and the first exon of *SFPQ*. (*I*) p53 binds to p53-RE-2, determined by the ChIP assay. *P < 0.05, **P < 0.01, ***P < 0.001.

The backsplicing circularization of circular RNAs is catalyzed by the spliceosomal machinery and modulated by intronic complementary sequences (ICSs) and RNA-binding proteins (RBPs) (23). We then sought to explore potential RBPs that are involved in the biogenesis of circASCC3. By searching RBPmap (24), we found that several RBPs might be responsible for the circularization of circASCC3 by binding to the flanking intronic regions. To test the hypothesis, we conducted an RNAi screen for RBPs that could regulate circASCC3 expression in CAL51 and HCT116 cells (*SI Appendix*, Fig. S3 *A* and *B*). As a consequence, we identified that ablation of SFPQ dramatically induced circASCC3 expression in both cell lines (Fig. 3 *A* and *B*). To validate these results, we employed two independent siRNAs to knock down SFPQ expression and consistently showed that SFPQ depletion significantly elevated the expression levels of circASCC3, but reduced the levels of ASCC3 mRNA (Fig. 3 *C* and *D*). These results suggested that SFPQ might play a role in inhibiting the circularization of circASCC3, thereby increasing the production of linear transcripts of *ASCC3*. It has been known that repetitive elements are the major ICSs, which contribute to the backsplicing and formation of circular RNA (23). We speculated that SFPQ might associate with these repetitive elements to prevent the circularization of circASCC3. Indeed, several potential SFPQ-binding sites were predicted within the repetitive elements on the flanking intronic regions (Fig. 3*E*). The RIP assay was then performed to confirm that SFPQ could associate with four of the six potential binding sites on both flanking regions of the exons 5 to 8 (Fig. 3*F*). Interestingly, we had noted that the DNA damage-inducing agents and Nutlin-3 could induce the expression of circASCC3 more markedly than ASCC3 mRNA (Fig. 2 *A*–*G* and *SI Appendix*, Fig. S2 *A* and *B*). These results suggested that p53 might also play a role in promoting the circularization of circASCC3. Thus, we tested whether p53 regulated the expression of SFPQ. By re-analyzing our microarray data, we found that p53 activation reduced SFPQ expression (*SI Appendix*, Fig. S3*C*). RT-qPCR analysis was performed to verified that Cisplatin and Nutlin-3 inhibited the expression of SFPQ (*SI Appendix*, Fig. S3 *D* and *E*), which could be partially reversed by p53 depletion (Fig. 3*G* and *SI Appendix*, Fig. S3 *F* and *G*). In addition, activation of p53 by Nutlin-3 or Cisplatin reduced SFPQ protein levels (*SI Appendix*, Fig. S3 *H* and *I*), whereas knockdown of p53 elevated SFPQ protein levels (*SI Appendix*, Fig. S3 *J* and *K*) in both CAL51 and HCT116 cells. Finally, three potential p53-REs around the *SFPQ* promoter region were identified (Fig. 3*H*) through p53MH algorithm (22), and p53-RE2 was validated as a binding site for p53 via the ChIP assay (Fig. 3*I*). Together, these results indicate that p53 may promote the circularization of circASCC3 by repressing the expression of SFPQ.

### Ectopic circASCC3 Promotes Tumor Resistance to Genotoxic Stress.

**Fig. 4.**
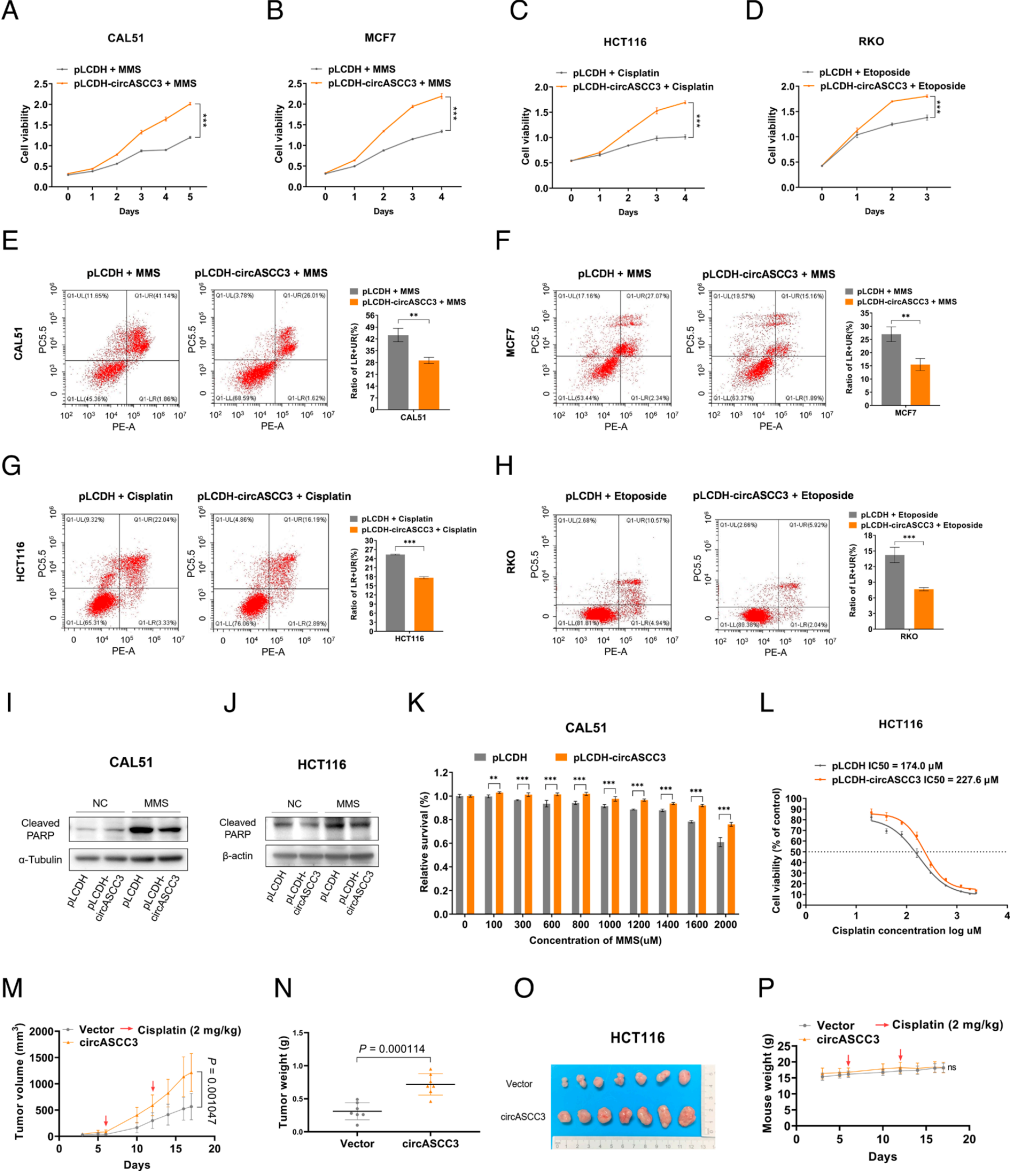
Ectopic circASCC3 promotes tumor resistance to genotoxic stress. (*A*–*D*) Overexpression of circASCC3 increases the growth of cancer cells exposed to the pulsed treatment with DNA damage-inducing agents. CAL51 (*A*), MCF7 (*B*), HCT116 (*C*), and RKO (*D*) cells were treated with MMS (1 mM), Cisplatin (100 μM), or Etoposide (80 μM) for 0.5 to 2 h, as illustrated in *SI Appendix*, Fig. S5*A*, and transfected with the indicated plasmids, followed by the cell viability assay. (*E*–*H*) Overexpression of circASCC3 reduces the apoptosis of cancer cells exposed to the pulsed treatment with DNA damage-inducing agents. (*I* and *J*) Overexpression of circASCC3 reduces the levels of cleaved PARP in cancer cells exposed to pulsed MMS. (*K* and *L*) Overexpression of circASCC3 increases the resistance of cancer cells that received pulsed treatments. (*M*–*P*) Stable overexpression of circASCC3 increases the growth rate (*M*), weight (*N*), and size (*O*) of HCT116-derived xenograft tumors. Mouse body weight is unaffected (*P*). All mice received Cisplatin treatment at Days 6 and 12 as indicated. Data are presented as mean ± SD, n = 7. *P* values were determined by two-tailed unpaired *t* test. **P < 0.01, ***P < 0.001.

To investigate the biological function of circASCC3, we ectopically overexpressed the circASCC3-encoding plasmid in various cancer cells (*SI Appendix*, Fig. S4 *A*–*D*). First, by conducting the cell viability assay and flow cytometric analysis, we found that circASCC3 overexpression could barely affect cell proliferation (*SI Appendix*, Fig. S4 *E*–*H*) or apoptosis (*SI Appendix*, Fig. S4 *I*–*L*). We then speculated that circASCC3 might play a role under DNA damage stress. To test this hypothesis, we conducted a pulse treatment of cancer cells, which closely resembles chemotherapy but minimizes extensive cell death (25), utilizing a strong DNA damage-inducing agent methyl methanesulfonate (MMS) as well as Cisplatin and Etoposide (*SI Appendix*, Fig. S5*A*). Unexpectedly, circASCC3 overexpression significantly promoted the growth of cancer cells when treated with these agents, as evidenced by the cell viability assay (Fig. 4 *A*–*D* and *SI Appendix*, Fig. S5 *B* and *C*). In addition, ectopic circASCC3 inhibited apoptosis upon genotoxic stress in various cancer cells, as indicated by the flow cytometric analysis (Fig. 4 *E*–*H* and *SI Appendix*, Fig. S5 *D*–*H*) and the levels of cleaved PARP (Fig. 4 *I* and *J* and *SI Appendix*, Fig. S5 *I* and *J*). Consistently, circASCC3 overexpression led to the resistance of cancer cells to MMS and Cisplatin (Fig. 4 *K* and *L*). Furthermore, our results showed that circASCC3 overexpression supported the growth of xenograft tumors treated with Cisplatin, as indicated by the increased tumor growth rate (Fig. 4*M*), weight (Fig. 4*N*), and size (Fig. 4*O*). The potential adverse events caused by the treatments were tolerable, as the average weight of the mice was not affected (Fig. 4*P*). Together, these results demonstrate that ectopic circASCC3 promotes cancer cell survival in vitro and in vivo under genotoxic stress.

### Ablation of circASCC3 Increases Tumor Sensitivity to Genotoxic Stress.

**Fig. 5.**
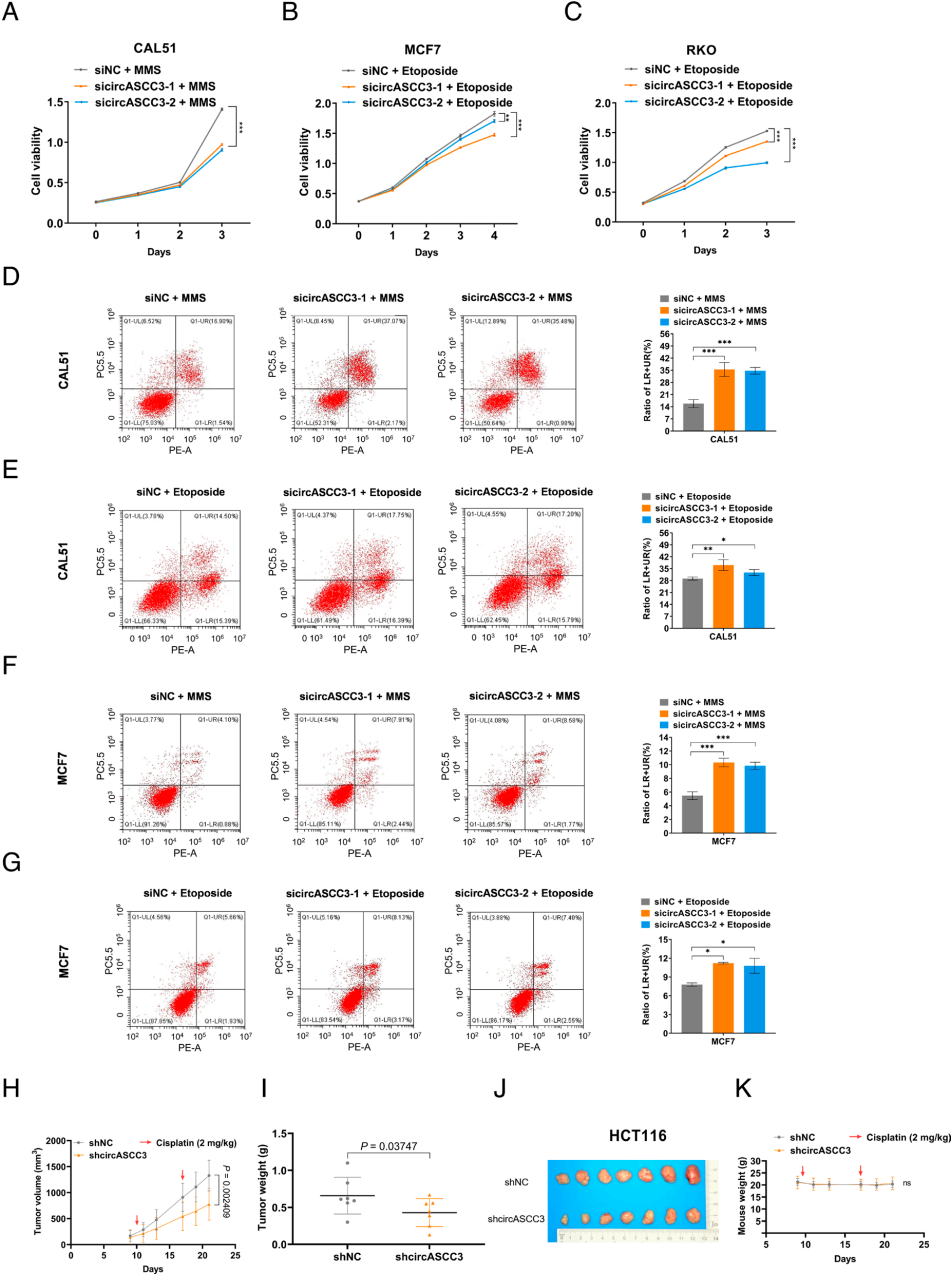
Ablation of circASCC3 increases tumor sensitivity to genotoxic stress. (*A*–*C*) Knockdown of circASCC3 suppresses the growth of cancer cells exposed to the pulsed treatment with DNA damage-inducing agents. (*D*–*G*) Knockdown of circASCC3 increases the apoptosis of cancer cells exposed to the pulsed treatment with DNA damage-inducing agents. (*H*–*K*) Stable knockdown of circASCC3 reduces the growth rate (*H*), weight (*I*), and size (*J*) of HCT116-derived xenograft tumors. Mouse body weight is unaffected (*K*). All mice received Cisplatin treatment at Days 10 and 17 as indicated. Data are presented as mean ± SD, n = 7. *P* values were determined by two-tailed unpaired *t* test. *P < 0.05, **P < 0.01, ***P < 0.001.

To examine the role of endogenous circASCC3, we depleted its expression using two independent siRNAs in various cancer cells, while the expression of *ASCC3* remained unaffected (*SI Appendix*, Fig. S6 *A*–*D*). Knockdown of circASCC3 barely affected cell growth (*SI Appendix*, Fig. S6 *E* and *F*) or apoptosis (*SI Appendix*, Fig. S6 *G* and *H*) in cancer cells when cultured in the unstressed condition. Nevertheless, depleting circASCC3 significantly suppressed cell growth (Fig. 5 *A*–*C*) and promoted apoptosis (Fig. 5 *D*–*G*) in cancer cells when treated with genotoxic agents. In line with these cell-based experiments, circASCC3 depletion inhibited the growth rate (Fig. 5*H*), weight (Fig. 5*I*), and size (Fig. 5*J*) of xenograft tumors treated with Cisplatin, without affecting the average weight of the mice (Fig. 5*K*). These results demonstrate that knockdown of circASCC3 enhances genotoxic stress-induced cell death in cancer.

It was reported that ASCC3 could collaborate with ALKBH3 to repair DNA alkylation damage in prostate and lung cancer cells that sustained high levels of ALKBH3 expression (26). Thus, we tested whether endogenous ASCC3 was involved in cell survival under genotoxic stress in our study. Under our experimental conditions, knockdown of ASCC3 by two different siRNAs (*SI Appendix*, Fig. S7 *A* and *B*) did not influence cell growth (*SI Appendix*, Fig. S7 *C* and *D*) or apoptosis (*SI Appendix*, Fig. S7 *E*–*H*) under both the unstressed and the genotoxic condition. These findings indicate that the function of circASCC3 in triggering resistance to genotoxic stress is independent of its host gene *ASCC3* in these cells.

### CircASCC3 Interacts with and Stabilizes DDX5.

**Fig. 6.**
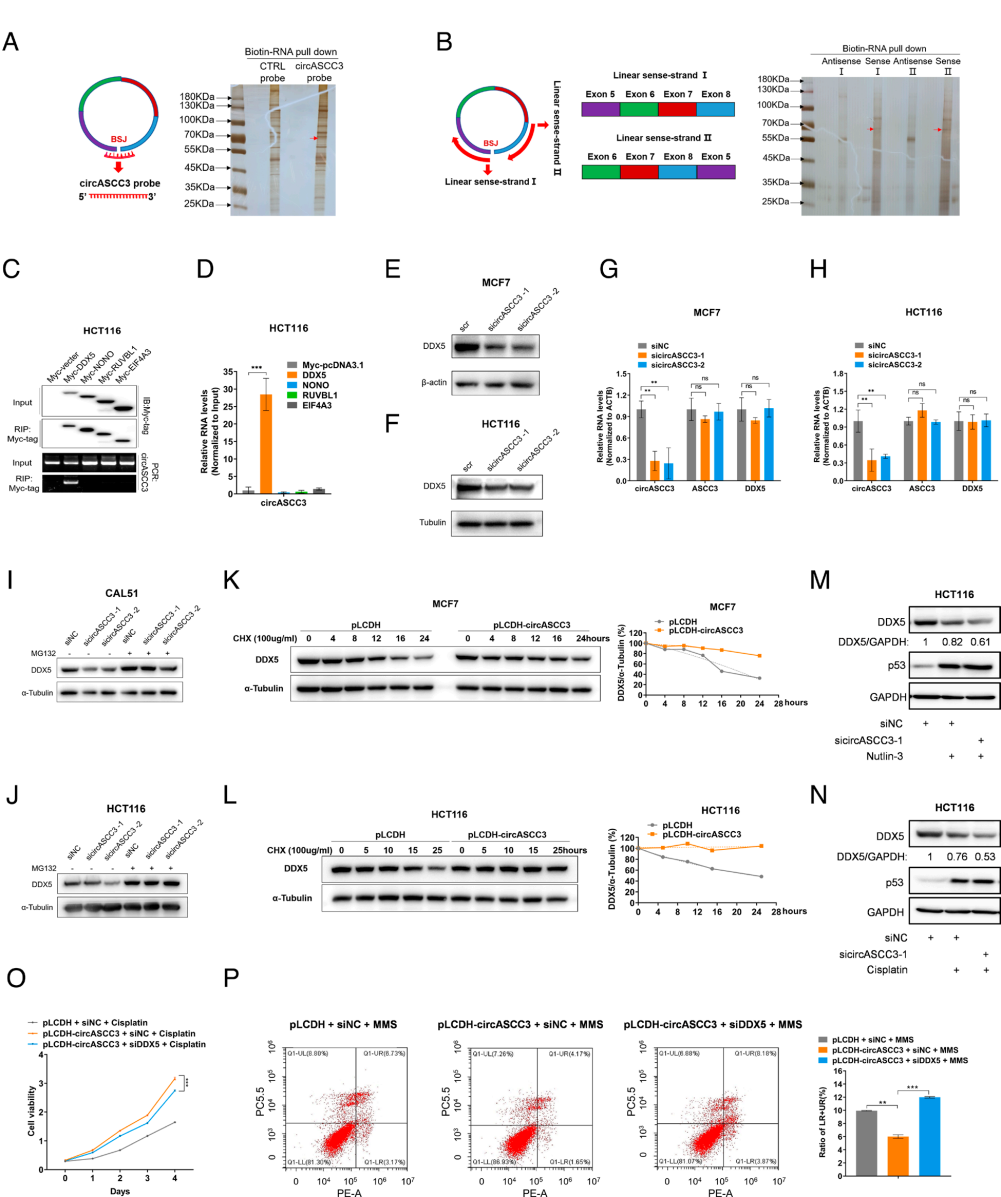
CircASCC3 interacts with and stabilizes DDX5. (*A*) Identification of circASCC3-interacting proteins using a biotinylated RNA probe complementary to the BSJ of endogenous circASCC3. (*B*) Identification of circASCC3-interacting proteins using biotinylated linear transcripts of circASCC3 as a bait. (*C* and *D*) CircASCC3 associates with DDX5, but not NONO, RUVBL1, or EIF4A3, determined by the RIP assay. (*E*–*H*) Knockdown of circASCC3 reduces the protein levels of DDX5, while not affecting mRNA expression of DDX5. (*I* and *J*) The proteasome inhibitor MG132 (20 μM) restores the protein levels of DDX5 in cells depleted of circASCC3. (*K* and *L*) Overexpression of circASCC3 prolongs the half-life of DDX5 protein. (*M* and *N*) Knockdown of circASCC3 further reduces DDX5 levels in response to Nutlin-3 (*M*) or Cisplatin (*N*) treatment. (*O* and *P*) Knockdown of DDX5 restores circASCC3-mediated cell growth and apoptosis. **P < 0.01, ***P < 0.001.

To understand the underlying mechanism by which circASCC3 supported cell survival in response to DNA damage stress, we conducted RNA-pull down assays using two strategies coupled with mass spectrometric (MS) analysis. CircASCC3-associated proteins were pulled down by a biotinylated, backsplicing junction (BSJ) probe against endogenous circASCC3 (Fig. 6*A*) and by the full-length, biotinylated, linearized circASCC3 transcripts (Fig. 6*B*), respectively. The protein complexes pulled down by both strategies were subjected to MS analysis, resulting in the identification of several proteins that might associate with circASCC3. RIP assays were then performed to validate that circASCC3 strongly bound to DDX5, as indicated by gel electrophoresis and RT-qPCR analyses (Fig. 6 *C* and *D*). However, there was no interaction observed with NONO, RUVBL1, or EIF4A3 (Fig. 6 *C* and *D*), despite the presence of these proteins in our MS results. DDX5 was shown to maintain genomic integrity and facilitate DNA damage repair by resolving R-loops (27–31). We thus speculated that circASCC3 might promote chemoresistance by regulating DDX5. Interestingly, we found that knockdown of circASCC3 reduced DDX5 protein levels (Fig. 6 *E* and *F*), while its mRNA expression remained unaffected (Fig. 6 *G* and *H*). This reduction could be completely restored in cancer cells when treated with the proteasome inhibitor MG132 (Fig. 6 *I* and *J*). Conversely, overexpression of circASCC3 extended the half-life of DDX5 protein (Fig. 6 *K* and *L*). In addition, we surprisingly found that Nutlin-3 or Cisplatin treatment led to a moderate reduction of DDX5 levels (Fig. 6 *M* and *N*), suggesting that the regulation of DDX5 by p53 is likely to be highly complex, particularly considering the fact that p53 can directly interact with DDX5 (32). Our results also showed that knockdown of circASCC3 could further reduce the levels of DDX5 in response to p53 activation (Fig. 6 *M* and *N*), which again indicates that p53-induced expression of circASCC3 is crucial for maintaining DDX5 protein stability. Moreover, our results showed that knockdown of DDX5 could partially reverse the effects of circASCC3 overexpression on cancer cell growth (Fig. 6*O*) and apoptosis (Fig. 6*P*). Together, these results suggest that circASCC3 triggers chemoresistance possibly by stabilizing DDX5 and thus eliminating R-loops, as elaborated below.

### CircASCC3 Represses R-loop Accumulation Through DDX5.

**Fig. 7.**
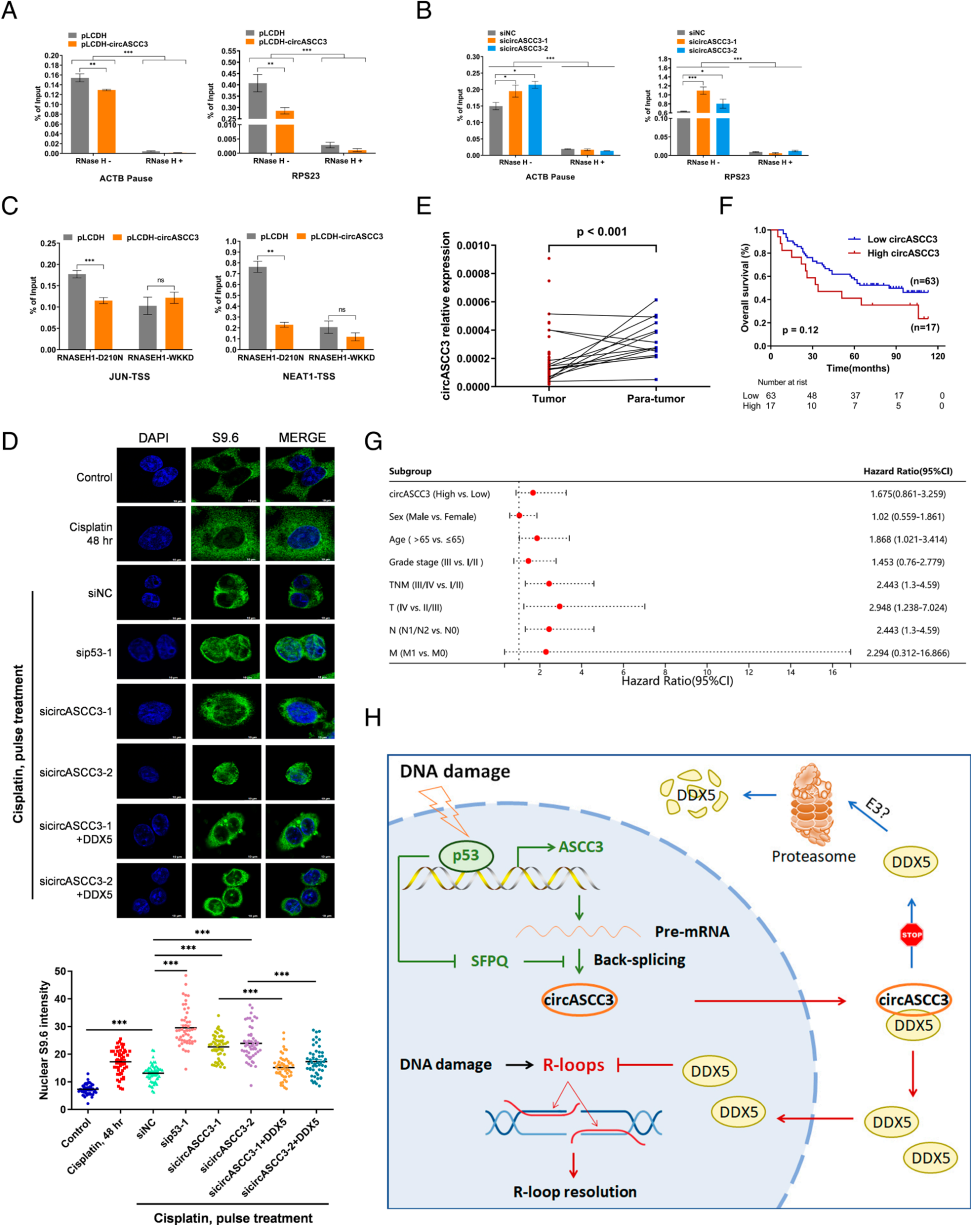
The p53–circASCC3 axis represses R-loop accumulation through DDX5. (*A*) Overexpression of circASCC3 reduces the level of R-loops formed at *ACTB* and *RPS23* loci, determined by the DRIP assay. (*B*) Knockdown of circASCC3 increases the level of R-loops formed at *ACTB* and *RPS23* loci, determined by the DRIP assay. RNase H serves as a negative control. (*C*) Overexpression of circASCC3 reduces the level of R-loops formed at *JUN* and *NEAT1* loci, determined by the R-ChIP assay. TSS, transcription start site. (*D*) Knockdown of circASCC3 increases the level of R-loops, while overexpression of DDX5 reverses this effect, determined by IF staining. (*E*) The levels of circASCC3 expression are lower in 15 colorectal cancer samples than matched adjacent normal tissues. (*F* and *G*) Higher levels of circASCC3 are associated with a worse prognosis in 80 colorectal cancer patients. (*H*) A schematic diagram for the role of the p53–circASCC3 axis in cancer. *P < 0.05, **P < 0.01, ***P < 0.001.

Since DDX5 restricts R-loops to maintain genomic stability (27–31), we examined whether circASCC3 played a role in resolving R-loops through DDX5 under DNA damage conditions. R-loops were found to accumulate at transcription pause sites, which are critical elements for transcription termination (33, 34). The human *ACTB* gene possesses a G-rich pause element, which could be utilized as a reference for indicating the formation of R-loops (34, 35). We performed DRIP-qPCR using the S9.6 antibody that specifically captures R-loops. Our results showed that overexpression of circASCC3 significantly reduced the level of R-loops formed at the *ACTB* pause site (Fig. 7*A*), whereas depletion of circASCC3 increased the formation of R-loops (Fig. 7*B*). These findings were further confirmed with an additional R-loop site at the *RPS23* locus (36) (Fig. 7 *A* and *B*). These results were reliable because R-loops could be almost eliminated by RNase H (Fig. 7 *A* and *B*), an endonuclease that selectively recognizes and resolves R-loops. An R-ChIP method was previously developed to detect R-loops using a catalytically inactive form of RNASEH1 (37). We then conducted the R-ChIP assay to test whether circASCC3 prevents R-loop accumulation. The D210N mutation of RNASEH1 was shown to bind to R-loops without breaking them down (37). Our results showed that overexpression of circASCC3 significantly reduced the levels of RNASEH1-D210N-bound R-loops (Fig. 7*C*). The WKKD mutant (W43A, K59A, K60A, and D210N) loses both catalytic and binding activities, which can be considered a negative control for comparison (37). Furthermore, S9.6 staining assay was performed to validate the function of circASCC3 in regulating R-loops. DNA damage induced by Cisplatin increased the levels of R-loops (Fig. 7*D*), which was consistent with previous studies (17, 38). Remarkably, knockdown of p53 or circASCC3 further promoted R-loop accumulation, while overexpression of DDX5 partially restored the R-loop levels (Fig. 7*D* and *SI Appendix*, Fig. S8*A*). Our results also revealed that SFPQ depletion significantly reduced R-loop levels, while the simultaneous knockdown of circASCC3 reversed the effect caused by SFPQ depletion (*SI Appendix*, Fig. S8 *B* and *C*). To determine whether p53 facilitates DNA damage repair, we evaluated the levels of γ-H2AX, a biomarker for double-strand breaks. As shown in *SI Appendix*, Fig. S9*A*, the treatment of cancer cells with a low dose of Nutlin-3 resulted in a more rapid decrease in γ-H2AX levels, demonstrating that p53 activation indeed promotes the repair of DNA damage caused by Cisplatin. Finally, we evaluated the clinical significance of circASCC3 in colorectal cancer samples (*SI Appendix*, Table S1). The levels of circASCC3 expression were lower in cancer tissues compared to normal tissues (Fig. 7*E*). This is possibly because p53 is usually inactivated or mutated in tumor cells. In line with its role in triggering tumor resistance to chemotherapy, higher levels of circASCC3 in tumors correlated with a worse prognosis (Fig. 7 *F* and *G* and *SI Appendix*, Table S2). However, the correlation was not statistically significant, and more samples may be needed for the analysis. Also, we observed a positive correlation between the expression levels of circASCC3 and DDX5 in ten colorectal cancer samples (*SI Appendix*, Fig. S9*B*). Collectively, our findings indicate that the p53–circASCC3 cascade maintains genomic stability by increasing DDX5-mediated resolution of R-loops.

## Discussion

p53 maintains genomic stability and suppresses cancer by regulating the transcription of a myriad of protein-coding and -noncoding genes. Recently, p53 was found to control the expression of circRNAs by activating the transcription of their host genes. Circ-MDM2 was the first circRNA identified to be regulated by p53 because it originates from the *MDM2* gene locus. Like MDM2, this circRNA promotes tumor cell growth by downregulating the protein level of p53 (39). IRSense is another p53-induced circRNA, which confers resistance to radiotherapy in lung cancer (40). Our study as presented here demonstrates that p53 activates the transcription of the host gene and facilitates the circularization of circASCC3 (Figs. 2 and 3).

The activation of p53 can suppress the development of cancer, but it may also lead to resistance to chemotherapy by enhancing DNA damage repair. Our findings suggest that p53-induced circASCC3 plays a role in the repair of DNA damage caused by chemotherapy. Mechanistically, circASCC3 interacts with and stabilizes DDX5 (Fig. 6), an RNA/DNA helicase that resolves R-loops. Although we showed that circASCC3 prevented the proteasomal degradation of DDX5, further investigation is needed to determine whether any E3 ligases are involved in this process. Interestingly, a recent study showed that circASCC3 promoted the growth of lung cancer cells even under normal growing conditions, suggesting that the regulatory mechanism of circASCC3 may vary in the context of different cancers (41). Finally, through multiple experimental approaches, we demonstrated that circASCC3 prevented R-loop accumulation through DDX5 under DNA damage stress (Fig. 7 *A*–*D*). We also noticed that Cisplatin-induced DNA damage significantly promoted R-loop accumulation (Fig. 7*D*), which was in line with previously studies (17, 38), while the basal level of R-loops was relatively low in cancer cells. This result may explain why circASCC3 has a marginal effect on the growth and apoptosis of cancer cells under normal growing conditions (*SI Appendix*, Figs. S4 and S6). By analyzing matched colorectal cancer samples, we found that circASCC3 was expressed at a higher level in normal tissues compared to cancer tissues (Fig. 7*E*), suggesting that circASCC3 may prevent the formation of cancer by maintaining genomic stability in normal cells. In addition, higher levels of circASCC3 in tumors predicted a worse prognosis (Fig. 7 *F* and *G*) possibly due to its role in resolving R-loops and conferring chemoresistance. Taken together, these findings uncover that circASCC3 plays a vital role in p53-mediated R-loop resolution and the maintenance of genomic stability.

Interestingly, our results reveal that the protein-coding gene *ASCC3* is a bona fide target gene of p53. This was because DNA damage-inducing agents or Nutlin-3 increased the expression of *ASCC3*, while knockdown of p53 reduced its expression (Fig. 2 *A*–*G*). Additionally, our ChIP and luciferase reporter assays verified that p53 associated with the *ASCC3* gene promoter to activate the transcription (Fig. 2 *P* and *Q*). The finding that p53 transcriptionally induces ASCC3 expression suggests a different mechanism for how p53 facilitates DNA damage repair. ASCC3 encodes a 3’-5’ DNA helicase, which coordinates with ALKBH3 to remove DNA alkylation adducts (26). It was found that ASCC3 conferred resistance to alkylation damage in ALKBH3-highly expressed PC3, LNCap, and H23 cells, whereas it had no impact on WiDr, U2OS, and HTB-1 cells with low levels of ALKBH3 expression (26). This study together with our findings indicates that p53 may be involved in the repair of alkylation damage in cancer cells with high levels of ALKBH3.

In conclusion, our study identifies a p53-inducible circular RNA circASCC3. Upon DNA damage stress, overexpression of circASCC3 increases the survival and growth of cancer cells, while depletion of circASCC3 inhibits their survival and growth. Mechanistically, circASCC3 interacts with and prevents the proteasomal degradation of DDX5, resulting in the resolution of R-loops and the consequent resistance to DNA damage (Fig. 7*H*). Our study unravels an important mechanism by which p53 maintains genomic integrity.

## Materials and Methods

Cells were cultured in DMEM supplemented with 10% FBS and 1% penicillin and streptomycin. Nude mice were obtained and maintained at Laboratory Animal Science of Fudan University Shanghai Cancer Center. Full details on the materials and methods are described in *SI Appendix*.

## Ethics Approval and Consent to Participate

The present study was approved by the ethics committee of the participating institutions.

## Supplementary Material

Appendix 01 (PDF)

## Data Availability

The microarray data has been deposited in the GEO database, with the accession number GSE253781 (42). All other data are included in the article and/or *SI Appendix*.
