# Supplementary material for: p53 activates circASCC3 to repress R-loops and enhance resistance to chemotherapy
Source: Proc Natl Acad Sci U S A. 2025 Mar 11;122(11):e2415869122. doi: 10.1073/pnas.2415869122 (PMC11929464; doi:10.1073/pnas.2415869122)
Supplement: Supplementary file 1 — Appendix 01 (PDF) [file pnas.2415869122.sapp.pdf]

PNAS

Supporting Information for

p53 activates circASCC3 to repress R-loops and enhance resistance to chemotherapy

Mingming Cao[1,2,#], Yu Gan[1,2,#], Yingdan Huang[1,2], Jing Tong[1,2], Chen Xiong[1,2], Yajie Chen[2,3], Bing Chen[1,2], Ruixuan Huang[4,5], Bangxiang Xie[6,7], Jun Deng[4,5], Shenglin Huang[1,2,8,9], Xianghuo He[1,2,8,9], Qian Hao[1,2,8,*], and Xiang Zhou[1,2,8,9,*]

[1] Fudan University Shanghai Cancer Center and Institutes of Biomedical Sciences, Fudan University, Shanghai 200032, China

[2] Department of Oncology, Shanghai Medical College, Fudan University, Shanghai 200032, China

[3] Department of Radiation Oncology, Fudan University Shanghai Cancer Center, Shanghai 200032, China

[4] Department of Oncology, The First Affiliated Hospital, Jiangxi Medical College, Nanchang University, Nanchang 330006, Jiangxi, China

[5] Jiangxi Key Laboratory for Individual Cancer Therapy, Nanchang 330006, Jiangxi, China

[6] Beijing Institute of Hepatology, Beijing Youan Hospital, Capital Medical University, Beijing 100069, China

[7] Beijing Engineering Research Center for Precision Medicine and Transformation of Hepatitis and Liver Cancer, Beijing 100069, China

[8] Key Laboratory of Breast Cancer in Shanghai, Fudan University Shanghai Cancer Center, Fudan University, Shanghai, 200032, China

[9] Shanghai Key Laboratory of Medical Epigenetics, International Co-laboratory of Medical Epigenetics and Metabolism (Ministry of Science and Technology), Institutes of Biomedical Sciences, Fudan University, Shanghai 200032, China

[#] Equal contribution

[*] Correspondence:

Qian Hao, Email: haoqian@fudan.edu.cn

Xiang Zhou, Email: xiangzhou@fudan.edu.cn

This PDF file includes:

Supporting text
SI References
Figures S1 to S9
Tables S1 to S11

Supporting Materials and Methods

Plasmids and antibodies

The loop sequence of circASCC3 was cloned into the plasmid pLCDH-GFP-CiR. DDX5, NONO, RUVBL1, and EIF4A3 were cloned into the Myc-tagged pcDNA3.1 vector. The p53-responsive element (p53-RE) was cloned into the pGL3 vector. The primers used for plasmid construction are listed in Supplementary Table S3. The shRNA oligos listed in Supplementary Table S4 were cloned into the pLKO.1 vector. The pPyCAG-RNASEH1-D210N and -WKKD plasmids were gifts from Liang Chen's lab. The anti-Flag (F1804, Sigma-Aldrich), anti-Myc (60003-2-Ig and 16286-1-AP, Proteintech), anti-DDX5 (Cat. No. 67025-1-Ig, Proteintech), anti-α-Tubulin (66031-1-Ig, Proteintech), anti-β-actin (Cat. No. 66009-1-Ig, Proteintech), anti-Cleaved PARP (Cat. No. 13371-1-AP, Proteintech), anti-γ-H2AX (Cat. No. 9718, Cell Signaling Technology), and anti-S9.6 (Cat. No. GS50001, Mabnus) were commercially purchased.

Cell culture and transient transfection

Human cancer cell lines, including CAL51, MCF7, HCT116, HCT116 $^{p53-/-}$, RKO, H1299, TOV112D, and ES-2, were cultured in DMEM supplemented with 10% FBS and 1% penicillin and streptomycin. OVCA420 cells were cultured in 1640 supplemented with 10% FBS and 1% penicillin and streptomycin. All cells were mycoplasma-free and authenticated by the Mycoplasma Detection Kit (Yeasen, Shanghai, China) and cultured at 37°C in a humidified incubator containing 5% CO_2. Plasmids and siRNAs were transiently transfected using Hieff Trans Liposomal transfection reagent according to the manufacture's protocol (Yeasen, Shanghai, China). SiRNAs used in the study are listed in Supplementary Table S5.

Quantitative real-time polymerase chain reaction

Total RNA was extracted using RNAiso Plus (Takara, Japan). Complementary DNA (cDNA) synthesis was performed using HiScript® IIIRT SuperMix for qPCR (+gDNA wiper) (Vazyme). RNA quantitative real-time polymerase chain reaction (RT-qPCR) was performed using SYBR Green SuperMix (Takara, Japan) and QuantStudio™ 6 Flex Real-Time PCR System (Thermo Fisher Scientific, USA). The primers used are listed in Supplementary Table S6.

RNA stability analysis

For Actinomycin D treatment, cells were seeded into six-well plates and grew up to 60% confluence. Cells were then treated with 5 μg/ml Actinomycin D and collected at the indicated time points. Total RNA was extracted using RNAiso Plus (Takara, Japan) and analyzed by RT-qPCR. For RNase R treatment, total RNA (3 μg) was incubated with 5U of RNase R (Lucigen, Wisconsin, USA) for 15 m at 37°C and then 70°C for 10 m to inactivate RNase R. The abundance of the indicated RNAs were analyzed by RT-qPCR. The primers used are listed in Supplementary Table S7.

RNA pull-down

Cell lysates were prepared in RIP buffer supplemented with complete protease inhibitor cocktail and RNase inhibitor. Cell lysates were incubated with the in vitro transcribed biotin-labelled RNA or RNA probes at 4°C for 3 h. The primers used for in vitro transcription are listed in Supplementary Table S8. The mixtures were then incubated with precleared streptavidin magnetic beads at 4°C overnight. After washing the mixtures for five times with RIP buffer, SDS loading buffer was added into beads and boiled at 100°C for 10 m. The samples were analyzed by SDS-PAGE gels and mass spectrum.

Immunoblotting

Immunoblotting (IB) was conducted as previously described (1). Briefly, cells were harvested and lysed in lysis buffer consisting of 50 mM Tris/HCl (pH8.0), 150 mM NaCl, 1% (v/v) Triton X-100, 0.1% (w/v) SDS, 1% sodium deoxycholate, and complete protease inhibitor cocktail. Equal amounts of protein lysates were resolved by an SDS-PAGE gel and then transferred on a PVDF membrane (Millipore, USA). The membrane was incubated with primary antibodies at 4°C overnight and then with a secondary antibody at room temperature for 1 h. The immunoreactive signals were visualized by an enhanced chemiluminescence (ECL) kit.

Immunoprecipitation

Cells were harvested and lysed in immunoprecipitation (IP) lysis buffer consisting of 50 mM Tris/HCl pH7.5, 150 mM NaCl, 5 mM EDTA, 0.5% (v/v) NP-40, and complete protease inhibitor cocktail. 500 to 1000 mg total proteins were incubated with the indicated antibodies at 4°C overnight and then mixed with protein A or G beads (Santa Cruz Biotechnology, USA) at 4°C for 2 h. The mixture was washed five times with IP lysis buffer. The bound proteins were detected by IB analysis.

Immunofluorescence staining

Cells were treated with or without Cisplatin and transfected with siRNAs and plasmids as indicated in the figure. Then, cells were fixed with methanol overnight at −20°C. Cells were

washed with PBS three times and blocked with the blocking buffer (8% BSA and 0.3% Triton X-100) for 1 h at room temperature. Next, cells were incubated with the primary antibody (anti-S9.6, 1:100) at 4°C overnight. Cells were then washed with PBS and incubated with the fluorescent secondary antibody (Yeasen) and DAPI (Sigma-Aldrich). Images were obtained with a confocal fluorescence microscope (Leica, Wetzlar, Germany).

RNA immunoprecipitation

Cells were lysed in RNA immunoprecipitation (RIP) buffer consisting of 10 mM Tris/HCl (pH7.4), 150 mM NaCl, 1 mM EDTA, 1 mM dithiothreitol, 0.1% (w/v) sodium dodecyl sulfate, 1% (v/v) NP-40, complete protease inhibitor cocktail, and RNase inhibitor. Equal cell lysates were immunoprecipitated with the indicated antibodies at 4℃ overnight before incubating with protein A or G beads at 4℃ for 3 h. 2% of each sample was used for input analysis by immunoblotting or PCR. After washing the mixtures for six times, bound RNAs were extracted using RNAiso Plus and analyzed by RT-qPCR. The primers used are listed in Supplementary Table S9.

Chromatin immunoprecipitation

Chromatin immunoprecipitation (ChIP) assay was performed using the Magna ChIP™ A/G Chromatin IP Kit following the manufacturer's protocol (Merck). Briefly, 1% formaldehyde was added to cell dishes to crosslink cells at room temperature for 10 m and then glycine was added to a final concentration of 125 mM to stop crosslinking. After rinsing with ice-cold PBS, cells were scraped from dishes and suspended in the ChIP lysis buffer supplemented with a protease inhibitor. Then cell pellets were collected and lysed using the nuclear lysis buffer. The chromatins were sheared into fragments ranging from 200 to 1000 bp by sonication. Magnetic beads with the anit-p53 antibody or IgG were mixed with the sheared chromatins at 4℃ overnight. Beads were washed four times and eluted using the elution buffer. Bound DNAs were purified and analyzed by qPCR. The primers used are listed in Supplementary Table S10.

DNA-RNA immunoprecipitation

Cells were lysed with SDS/proteinase K at 37°C overnight. Nucleic acids were purified using phenol-chloroform and High Density Maxtract Phase Lock Gel, followed by ethanol precipitation at room temperature. The nucleic acids obtained were digested overnight at 37°C using a combination of restriction enzymes (BsrGI, EcoRI, HindIII, SspI, and XbaI) in NEB buffer 2.1 supplemented with 1 mM Spermidine and 100 μg/ml BSA. Digested DNAs were purified through phenol-chloroform extraction using High Density Maxtract Phase Lock Gel and then either treated with RNase H overnight at 37°C in NEB RNase H buffer or left untreated. DNA:RNA hybrids from 10 μg digested nucleic acids were incubated with 3 μl S9.6 antibody, which specifically captures DNA:RNA hybrids, and 50 μl protein A/G agarose beads at 4°C for 3 h in DNA-RNA immunoprecipitation (DRIP) binding buffer (10 mM $NaPO_4$, 140 mM NaCl,

0.05% Triton X-100). Beads were then washed three times with DRIP binding buffer at room temperature, and eluted with the elution buffer (50 mM Tris pH 8.0, 10 mM EDTA pH 8.0 and 0.5% SDS, and 140 μg protease K) at 55°C for 45 m. The nucleic acids were purified through phenol-chloroform extraction, followed by ethanol precipitation at -20°C overnight. Quantitative PCR was performed using the primers listed in Supplementary Table S11.

R-chromatin immunoprecipitation

The R-chromatin immunoprecipitation (R-ChIP) assay was described previously (2). Briefly, HEK-293T cells expressing V5-tagged RNASEH1-D210N or -WKKD mutant protein were crosslinked with 1% formaldehyde for 10-15 m at room temperature. Glycine was added to a final concentration of 125 mM for 15 m at room temperature to stop the reaction. After washing twice with PBS, cells were scraped off and the nuclei were extracted using cell lysis buffer (10 mM Tris/HCl pH8.0, 10 mM NaCl, 0.5% Igepal CA-630) and suspended in nuclear lysis buffer (50 mM Tris/HCl pH8.0, 10 mM EDTA pH8.0, 1% SDS). Chromatin DNA was fragmented into 100-600 bp fragments using sonication. 5% of chromatin fragments were reserved for input analysis. The remaining samples were incubated with beads conjugated with the anti-V5 antibody at 4°C overnight. Beads were then washed three times with wash buffer I (20 mM Tris/HCl pH8.0, 2 mM EDTA pH8.0, 1% Triton X-100, 0.1% SDS, 150 mM NaCl), three times with wash buffer II (20 mM Tris/HCl pH8.0, 2 mM EDTA pH8.0, 1% Triton X-100, 0.1% SDS, 500 mM NaCl), once with wash buffer III (10 mM Tris/HCl pH8.0, 1 mM EDTA pH8.0, 1% Igepal CA-630, 250 mM LiCl, 1% sodium deoxycholate), and once with TE buffer. Then the beads were incubated with elution buffer (10 mM Tris/HCl pH8.0, 1 mM EDTA pH8.0, 1% SDS) at 65°C overnight and treated with RNase A and Proteinase K. The DNAs were purified through phenol-chloroform extraction and ethanol precipitation at -20°C overnight. The samples were subjected to PCR analysis using primers listed in Supplementary Table S11.

Cell viability assay

The Cell Counting Kit-8 (CCK-8) (Dojindo, Shanghai, China) was used for the cell viability assay according to the manufacturer's instructions. Cells transfected with the indicated plasmids or siRNAs or treated with pulsed agents were seeded in 96-well culture plates at a density of 2000-4000 cells per well. 10% WST-8 was added to each well every 24 h, and the absorbance of the samples was measured at 450 nm.

Flow cytometry

Cell apoptosis was assessed using the PE annexin V apoptosis detection kit (BD Pharmingen). Cells were harvested with trypsin, washed with precooled PBS, and resuspended with 100 μl 1 × binding buffer. Cells were then incubated with Annexin V-PE and 7-aminoactinomycin D (7AAD) at room temperature for 15 m in the dark. The level of apoptosis was determined by flow cytometry (CytoFLEX S, Beckman Coulter).

Mouse xenograft experiment

Five-week-old female BALB/c nude mice were obtained from Laboratory Animal Science of Fudan University Shanghai Cancer Center. 4×10^6 HCT116 cells stably overexpressing circASCC3 or shcircASCC3 or control vectors were resuspended in 100 μl serum-free medium and subcutaneously injected into flank regions of mice. Tumor growth was monitored every other day with electronic digital vernier caliper in two dimensions. Tumor volume was calculated according to the formula: volume = length × width^2 × 0.52. When the tumors reached an appropriate volume, they were harvested, weighted, and photographed. The animal protocols were in compliance with ethical guidelines and approved by the Animal Welfare Committee of Fudan University Shanghai Cancer Center.

Whole-transcriptome microarray

CAL51 cells were treated with DMSO, Nutlin-3, 5-fluorouracil, or Cisplatin for 48 h. Total RNA was extracted using RNAiso Plus (Takara, Japan). Microarray analysis was provided by Shanghai Biotechnology Corporation (Shanghai, China).

Colorectal cancer specimens

A total of 80 colorectal cancer tissues with 15 matched adjacent normal tissues were used to construct a cDNA microarray (Shanghai Outdo Biotech, Shanghai, China), which was subjected to qPCR analysis of the expression of circASCC3. This study was approved by the Human Research Ethics Committee of Fudan University Shanghai Cancer Center.

Statistical analysis

All in vitro experiments were conducted in biological triplicate. Differences between two or more groups were analyzed by the Student's t-test or one-way ANOVA. Statistical analyses were conducted using GraphPad Prism 8.0 and presented as means ± the standard deviation (SD). Kaplan-Meier plot method and log-rank tests were used to analyze significant differences in patient survival. Asterisks denote statistical significance: * $p < 0.05$; ** $p < 0.01$; *** $p < 0.001$.

References

1. Y. Huang *et al.*, p53-responsive CMBL reprograms glucose metabolism and suppresses cancer development by destabilizing phosphofructokinase PFKP. *Cell reports* **42**, 113426 (2023).
2. J. Y. Chen, X. Zhang, X. D. Fu, L. Chen, R-ChIP for genome-wide mapping of R-loops by using catalytically inactive RNASEH1. *Nature protocols* **14**, 1661-1685 (2019).

Supplementary Figure Legends

Figure S1. Identification of circRNAs that are responsive to chemotherapeutic agents. Related to Figure 1.

(A) The volcano plot of genes that are differentially expressed upon the treatments with all three agents (Nutlin-3, Cisplatin, and 5-FU). **(B-D)** RT-qPCR analysis of the expression of circRNAs in cancer cells when treated with DMSO, Nutlin-3, Cisplatin, or 5-FU. **(E)** CircASCC3 is mainly localized to the cytoplasm in RKO cells.

Figure S2. p53 regulates the expression of circASCC3. Related to Figure 2.

(A) The expression of circASCC3 and ASCC3 mRNA is elevated upon Cisplatin treatment in RKO cells. Right panels indicate relative abundance of circASCC3 by normalizing to ASCC3 mRNA. **(B)** Knockdown of p53 abolishes the elevation of both circASCC3 and ASCC3 mRNA levels in Hey cells. **(C, D)** Knockdown of p53 using a second siRNA abolishes the elevation of both circASCC3 and ASCC3 mRNA levels in HCT116 and CAL51 cells. ** $p < 0.01$, *** $p < 0.001$.

Figure S3. Identification of SFPQ as a regulator of circASCC3. Related to Figure 3.

(A, B) The efficiency of knocking down the expression levels of a panel of RNA-binding proteins. **(C)** The volcano plot reveals that SFPQ is downregulated in cancer cells when treated with Nutlin-3, Cisplatin, or 5-FU. **(D, E)** SFPQ mRNA levels are reduced upon Nutlin-3 or Cisplatin treatment. **(F, G)** Knockdown of p53 by two independent siRNAs elevates the levels of SFPQ mRNA. **(H, I)** Nutlin-3 or Cisplatin treatment reduces SFPQ protein levels. **(J, K)** Knockdown of p53 elevates SFPQ protein levels. * $p < 0.05$, ** $p < 0.01$, *** $p < 0.001$.

Figure S4. Ectopic circASCC3 has no impact on the growth of cancer cells under normal growing conditions. Related to Figure 4.

(A-D) The efficiency of circASCC3 overexpression in different cancer cell lines. **(E-H)** Overexpression of circASCC3 does not affect the growth of cancer cells under normal growing conditions. **(I-L)** Overexpression of circASCC3 does not affect the apoptosis of cancer cells under normal growing conditions. *** $p < 0.001$.

Figure S5. Ectopic circASCC3 promotes tumor resistance to genotoxic stress. Related to Figure 4.

(A) The experimental design of the pulse treatment of cells with DNA damage-inducing agents. **(B, C)** Overexpression of circASCC3 increases the growth of cancer cells treated with pulsed Etoposide or MMS. **(D-H)** Overexpression of circASCC3 reduces the apoptosis of cancer cells treated with pulsed Cisplatin, Etoposide, or MMS. **(I, J)** Overexpression of circASCC3 reduces the levels of cleaved PARP in cancer cells treated with pulsed Cisplatin or Etoposide. * p <

0.05, ** p < 0.01, *** p < 0.001.

Figure S6. Ablation of circASCC3 has no impact on the growth of cancer cells under normal growing conditions. Related to Figure 5.

(A-D) The efficiency of circASCC3 knockdown in different cancer cell lines. **(E, F)** Knockdown of circASCC3 does not affect the growth of cancer cells under normal growing conditions. **(G, H)** Knockdown of circASCC3 does not affect the apoptosis of cancer cells under normal growing conditions. * p < 0.05, ** p < 0.01, *** p < 0.001.

Figure S7. Ablation of ASCC3 has no impact on the growth of cancer cells.

(A, B) The efficiency of ASCC3 knockdown in different cancer cell lines. **(C, D)** Knockdown of ASCC3 does not significantly affect the growth of cancer cells. **(E-H)** Knockdown of ASCC3 does not significantly affect the apoptosis of cancer cells. *** p < 0.001.

Figure S8. R-loop levels determined by S9.6 staining. Related to Figure 7.

(A) Knockdown of p53 by two independent siRNAs increases the level of R-loops as determined by S9.6 staining, which can be depleted by the overexpression of RNASEH1. **(B, C)** Knockdown of SFPQ reduces the level of R-loops caused by pulsed Cisplatin, while concurrent knockdown of circASCC3 reverses this effect. *** p < 0.001.

Figure S9. The p53-circASCC3-DDX5 axis play a role in DNA damage repair. Related to Fgiure 7.

(A) Nultin-3-induced p53 activation promotes the repair of DNA damage caused by pulsed Cisplatin. HCT116 cells were treated with pulsed Cisplatin (200 μM) for 45 m, after which the medium was replaced with a fresh one. Nutlin-3 (5 μM) and Z-VAD-FMK (15 μM) were then added to the medium as indicated. Cells were collected for immunoblotting analysis at the timepoints as indicated. To observe DNA damage repair over an extended period, all cells were treated with the apoptosis inhibitor Z-VAD-FMK. **(B)** The expression levels of circASCC3 and DDX5 are positively correlated in ten colorectal cancer samples.

Figure S1

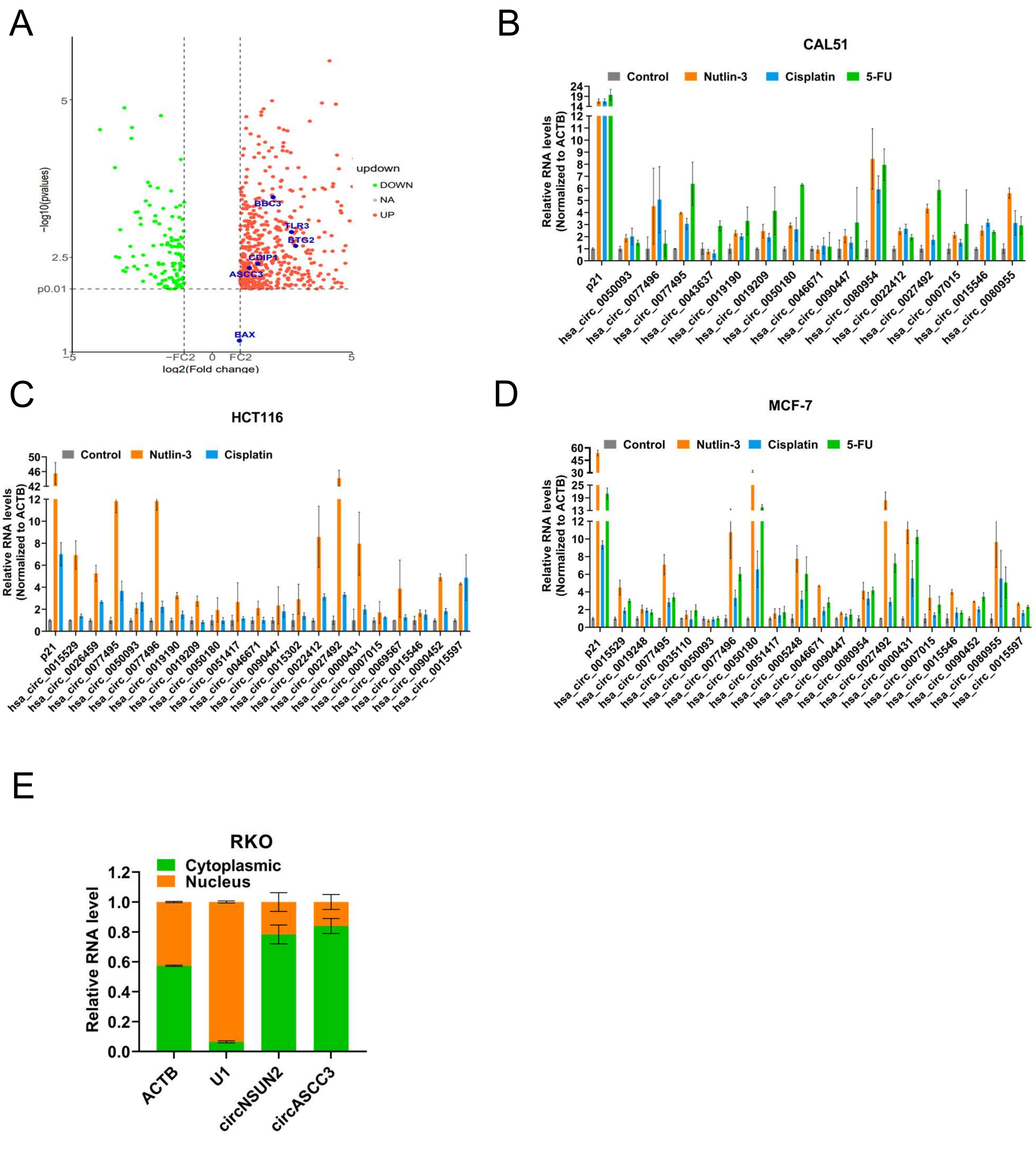
A
updown
DOWN
NA
UP
-log10(pvalues)
log2(Fold change)
p0.01
-FC2
FC2
BBC3
TLR3
BTG2
CDIP1
ASCC3
BAX
B
CAL51
Control
Nutlin-3
Cisplatin
5-FU
Relative RNA levels (Normalized to ACTB)
p21
hsa_circ_0050093
hsa_circ_0077496
hsa_circ_0077495
hsa_circ_0043637
hsa_circ_0019190
hsa_circ_0019209
hsa_circ_0050180
hsa_circ_0046671
hsa_circ_0090447
hsa_circ_0080954
hsa_circ_0022412
hsa_circ_0027492
hsa_circ_0007015
hsa_circ_0015546
hsa_circ_0080955
C
HCT116
Control
Nutlin-3
Cisplatin
Relative RNA levels (Normalized to ACTB)
p21
hsa_circ_0015529
hsa_circ_0026459
hsa_circ_0077495
hsa_circ_0050093
hsa_circ_0077496
hsa_circ_0019190
hsa_circ_0019209
hsa_circ_0050180
hsa_circ_0051417
hsa_circ_0046671
hsa_circ_0090447
hsa_circ_0015302
hsa_circ_0022412
hsa_circ_0027492
hsa_circ_0000431
hsa_circ_0007015
hsa_circ_0069567
hsa_circ_0015546
hsa_circ_0090452
hsa_circ_0015597
D
MCF-7
Control
Nutlin-3
Cisplatin
5-FU
Relative RNA levels (Normalized to ACTB)
p21
hsa_circ_0015529
hsa_circ_0019248
hsa_circ_0077495
hsa_circ_0035110
hsa_circ_0050093
hsa_circ_0077496
hsa_circ_0050180
hsa_circ_0051417
hsa_circ_0005248
hsa_circ_0046671
hsa_circ_0090447
hsa_circ_0080954
hsa_circ_0027492
hsa_circ_0000431
hsa_circ_0007015
hsa_circ_0015546
hsa_circ_0090452
hsa_circ_0080955
hsa_circ_0015597
E
RKO
Cytoplasmic
Nucleus
Relative RNA level
ACTB
U1
circNSUN2
circASCC3

Figure S2

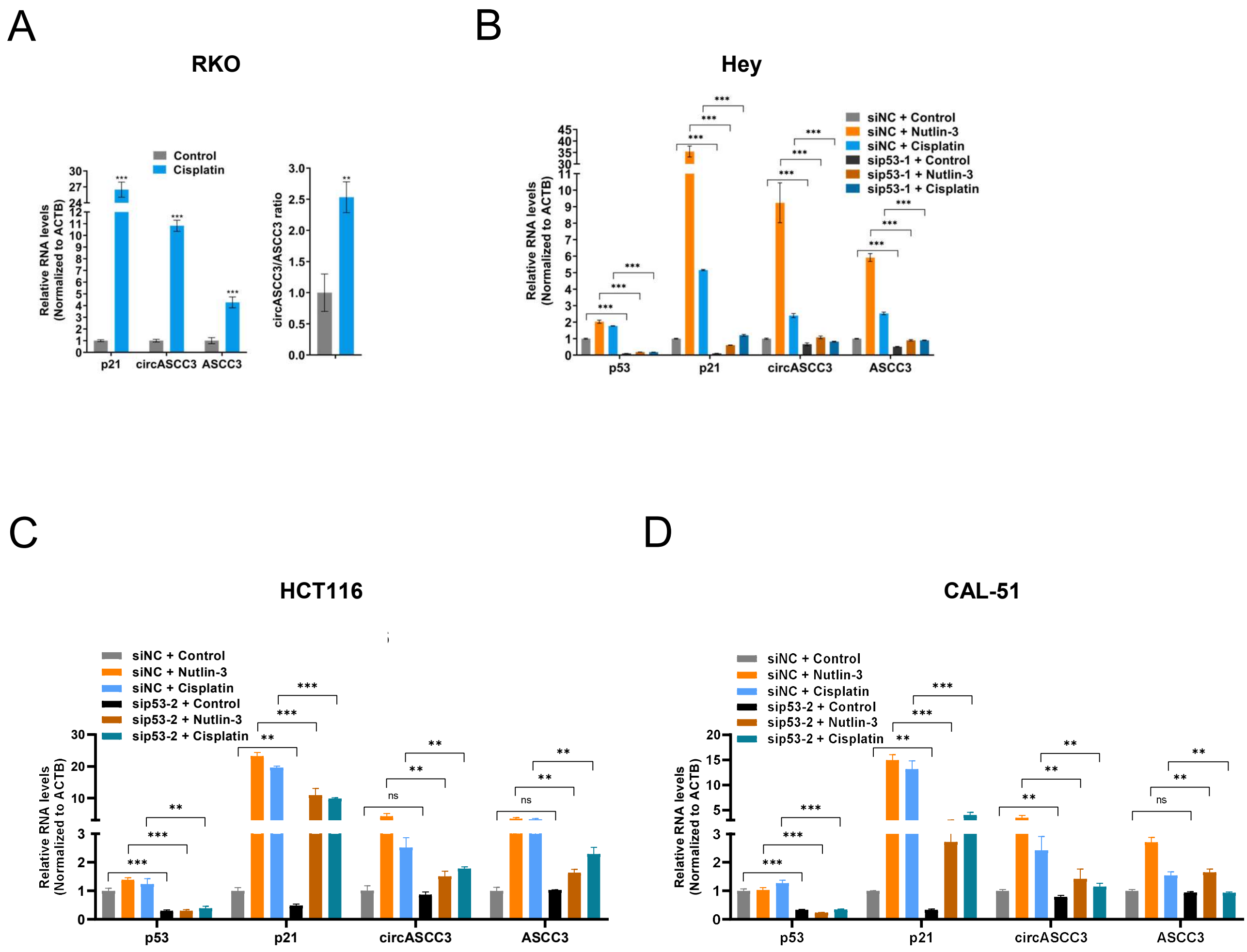
A
RKO
Control
Cisplatin
Relative RNA levels (Normalized to ACTB)
p21
circASCC3
ASCC3
circASCC3/ASCC3 ratio
B
Hey
siNC + Control
siNC + Nutlin-3
siNC + Cisplatin
sip53-1 + Control
sip53-1 + Nutlin-3
sip53-1 + Cisplatin
Relative RNA levels (Normalized to ACTB)
p53
p21
circASCC3
ASCC3
C
HCT116
siNC + Control
siNC + Nutlin-3
siNC + Cisplatin
sip53-2 + Control
sip53-2 + Nutlin-3
sip53-2 + Cisplatin
Relative RNA levels (Normalized to ACTB)
p53
p21
circASCC3
ASCC3
D
CAL-51
siNC + Control
siNC + Nutlin-3
siNC + Cisplatin
sip53-2 + Control
sip53-2 + Nutlin-3
sip53-2 + Cisplatin
Relative RNA levels (Normalized to ACTB)
p53
p21
circASCC3
ASCC3

Figure S3

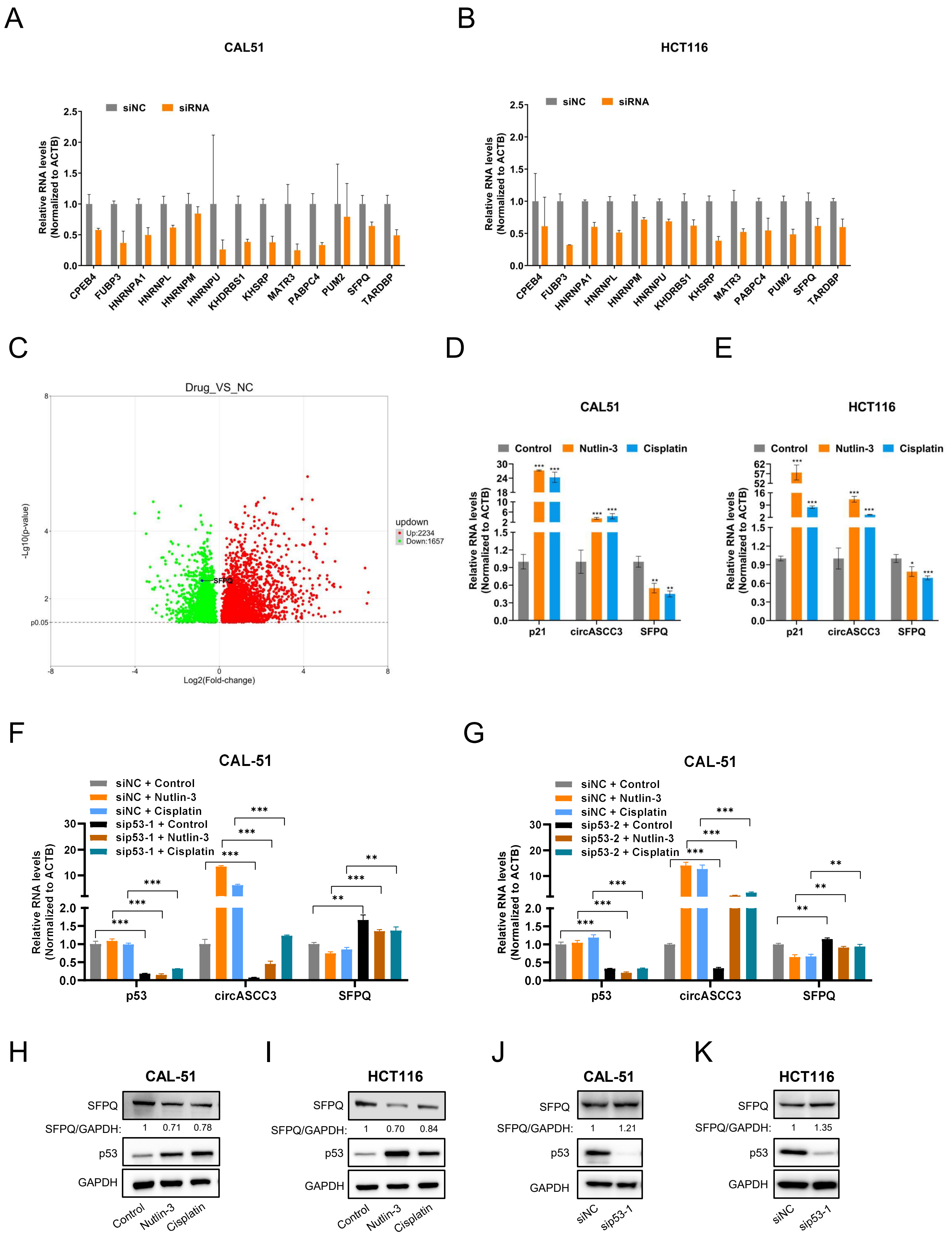
A
CAL51
siNC
siRNA
Relative RNA levels (Normalized to ACTB)
CPEB4
FUBP3
HNRNPA1
HNRNPL
HNRNPM
HNRNPU
KHDRBS1
KHSRP
MATR3
PABPC4
PUM2
SFPQ
TARDBP
B
HCT116
C
Drug_VS_NC
-Lg10(p-value)
Log2(Fold-change)
p0.05
SFPQ
updown
Up:2234
Down:1657
D
CAL51
E
HCT116
Control
Nutlin-3
Cisplatin
p21
circASCC3
SFPQ
F
CAL-51
siNC + Control
siNC + Nutlin-3
siNC + Cisplatin
sip53-1 + Control
sip53-1 + Nutlin-3
sip53-1 + Cisplatin
p53
G
CAL-51
sip53-2 + Control
sip53-2 + Nutlin-3
sip53-2 + Cisplatin
H
CAL-51
SFPQ
SFPQ/GAPDH: 1 0.71 0.78
p53
GAPDH
Control
Nutlin-3
Cisplatin
I
HCT116
SFPQ/GAPDH: 1 0.70 0.84
J
CAL-51
SFPQ/GAPDH: 1 1.21
siNC
sip53-1
K
HCT116
SFPQ/GAPDH: 1 1.35

Figure S4

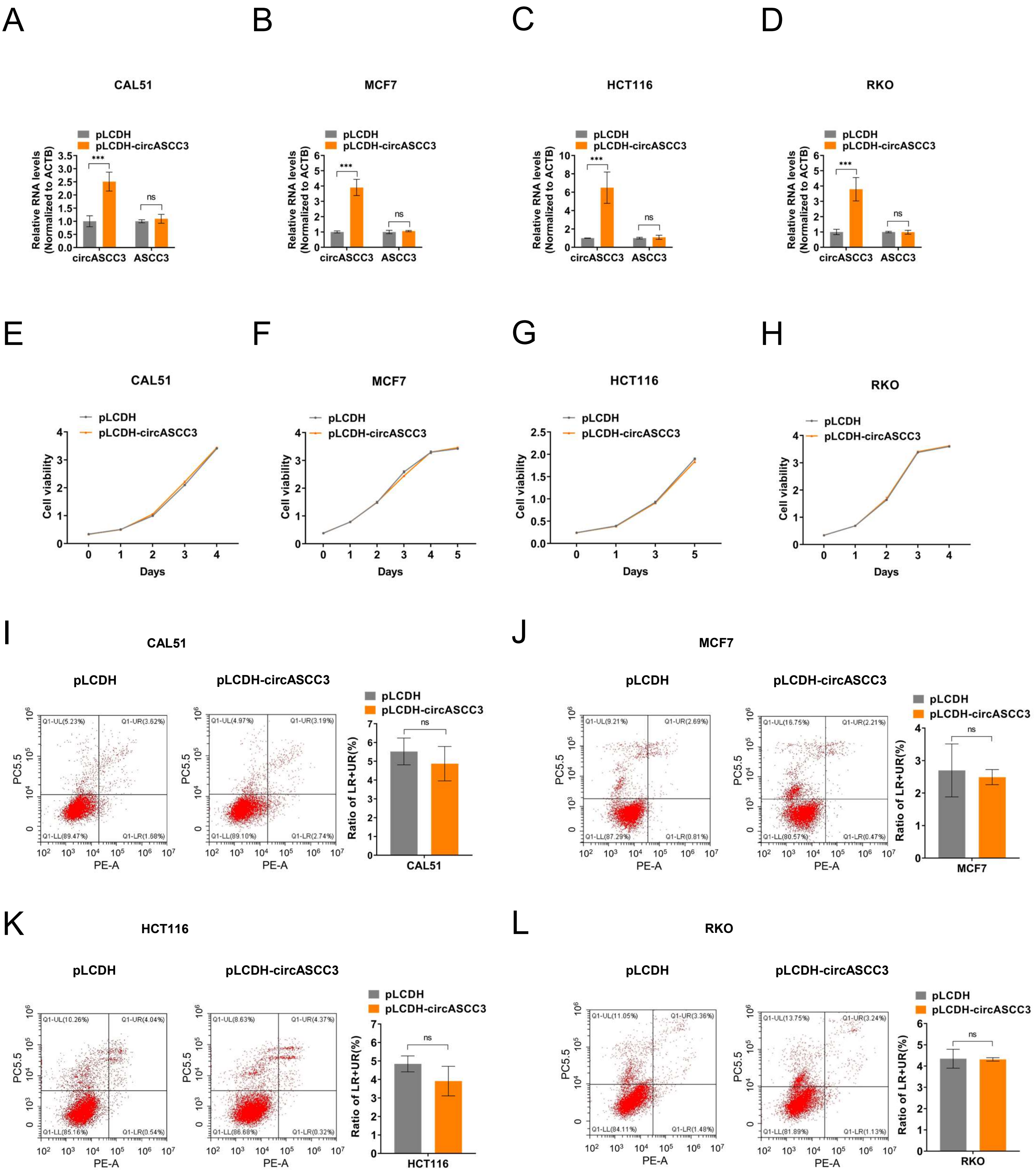

A
B
C
D
CAL51
MCF7
HCT116
RKO
pLCDH
pLCDH-circASCC3
Relative RNA levels (Normalized to ACTB)
***
ns
circASCC3
ASCC3
E
F
G
H
Cell viability
Days
I
J
K
L
PC5.5
PE-A
Ratio of LR+UR(%)
Q1-UL(5.23%)
Q1-UR(3.62%)
Q1-LL(89.47%)
Q1-LR(1.68%)
Q1-UL(4.97%)
Q1-UR(3.19%)
Q1-LL(89.10%)
Q1-LR(2.74%)
Q1-UL(9.21%)
Q1-UR(2.69%)
Q1-LL(87.29%)
Q1-LR(0.81%)
Q1-UL(16.75%)
Q1-UR(2.21%)
Q1-LL(80.57%)
Q1-LR(0.47%)
Q1-UL(10.26%)
Q1-UR(4.04%)
Q1-LL(85.16%)
Q1-LR(0.54%)
Q1-UL(8.63%)
Q1-UR(4.37%)
Q1-LL(86.68%)
Q1-LR(0.32%)
Q1-UL(11.05%)
Q1-UR(3.36%)
Q1-LL(84.11%)
Q1-LR(1.48%)
Q1-UL(13.75%)
Q1-UR(3.24%)
Q1-LL(81.89%)
Q1-LR(1.13%)

Figure S5

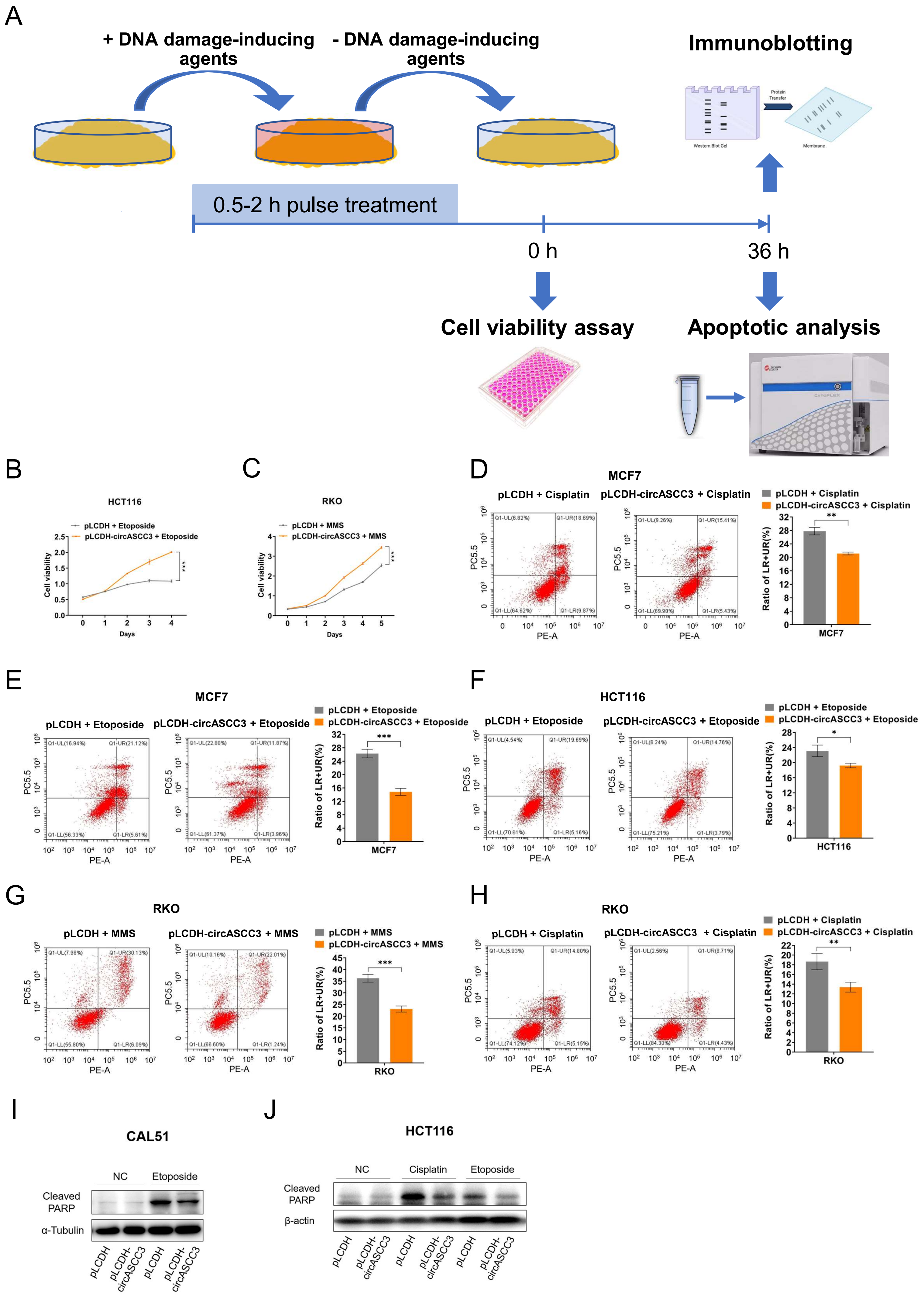

A
+ DNA damage-inducing agents
- DNA damage-inducing agents
Immunoblotting
Protein Transfer
Western Blot Gel
Membrane
0.5-2 h pulse treatment
0 h
36 h
Cell viability assay
Apoptotic analysis
B
HCT116
pLCDH + Etoposide
pLCDH-circASCC3 + Etoposide
Cell viability
Days
***
C
RKO
pLCDH + MMS
pLCDH-circASCC3 + MMS
Cell viability
Days
***
D
MCF7
pLCDH + Cisplatin
pLCDH-circASCC3 + Cisplatin
PC5.5
PE-A
Q1-UL(6.82%)
Q1-UR(18.69%)
Q1-LL(64.62%)
Q1-LR(9.87%)
Q1-UL(9.26%)
Q1-UR(15.41%)
Q1-LL(69.90%)
Q1-LR(5.43%)
Ratio of LR+UR(%)
**
MCF7
E
MCF7
pLCDH + Etoposide
pLCDH-circASCC3 + Etoposide
Q1-UL(16.94%)
Q1-UR(21.12%)
Q1-LL(56.33%)
Q1-LR(5.61%)
Q1-UL(22.80%)
Q1-UR(11.87%)
Q1-LL(61.37%)
Q1-LR(3.96%)
Ratio of LR+UR(%)
***
MCF7
F
HCT116
pLCDH + Etoposide
pLCDH-circASCC3 + Etoposide
Q1-UL(4.54%)
Q1-UR(19.69%)
Q1-LL(70.61%)
Q1-LR(5.16%)
Q1-UL(6.24%)
Q1-UR(14.76%)
Q1-LL(75.21%)
Q1-LR(3.79%)
Ratio of LR+UR(%)
*
HCT116
G
RKO
pLCDH + MMS
pLCDH-circASCC3 + MMS
Q1-UL(7.98%)
Q1-UR(30.13%)
Q1-LL(55.80%)
Q1-LR(6.09%)
Q1-UL(10.16%)
Q1-UR(22.01%)
Q1-LL(66.60%)
Q1-LR(1.24%)
Ratio of LR+UR(%)
***
RKO
H
RKO
pLCDH + Cisplatin
pLCDH-circASCC3 + Cisplatin
Q1-UL(5.93%)
Q1-UR(14.80%)
Q1-LL(74.12%)
Q1-LR(5.15%)
Q1-UL(2.56%)
Q1-UR(8.71%)
Q1-LL(84.30%)
Q1-LR(4.43%)
Ratio of LR+UR(%)
**
RKO
I
CAL51
NC
Etoposide
Cleaved PARP
α-Tubulin
pLCDH
pLCDH-circASCC3
pLCDH
pLCDH-circASCC3
J
HCT116
NC
Cisplatin
Etoposide
Cleaved PARP
β-actin
pLCDH
pLCDH-circASCC3
pLCDH
pLCDH-circASCC3
pLCDH
pLCDH-circASCC3

Figure S6

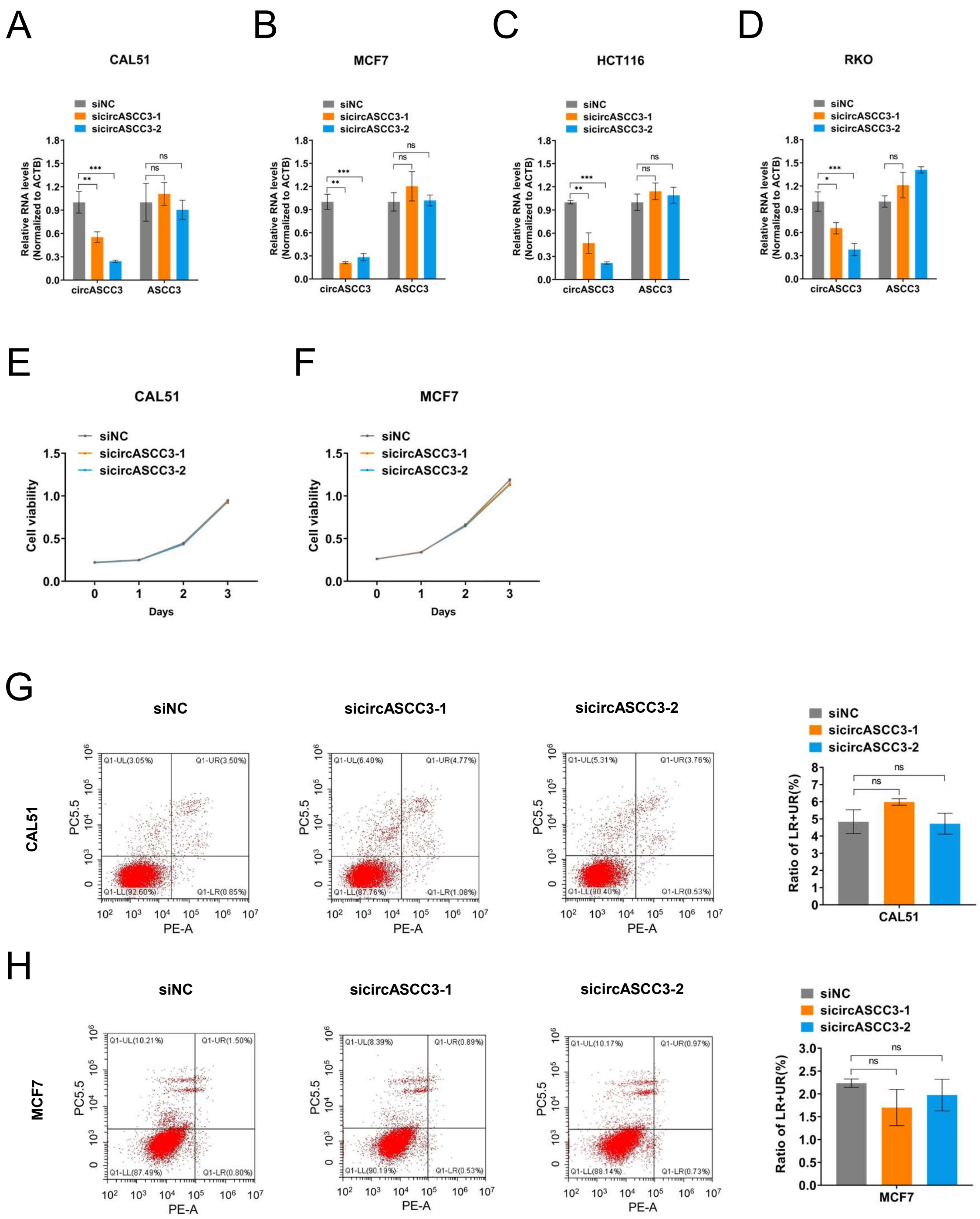

A
CAL51
B
MCF7
C
HCT116
D
RKO
siNC
sicircASCC3-1
sicircASCC3-2
Relative RNA levels (Normalized to ACTB)
circASCC3
ASCC3
ns
E
CAL51
F
MCF7
Cell viability
Days
G
siNC
sicircASCC3-1
sicircASCC3-2
CAL51
PC5.5
PE-A
Ratio of LR+UR(%)
H
MCF7

Figure S7

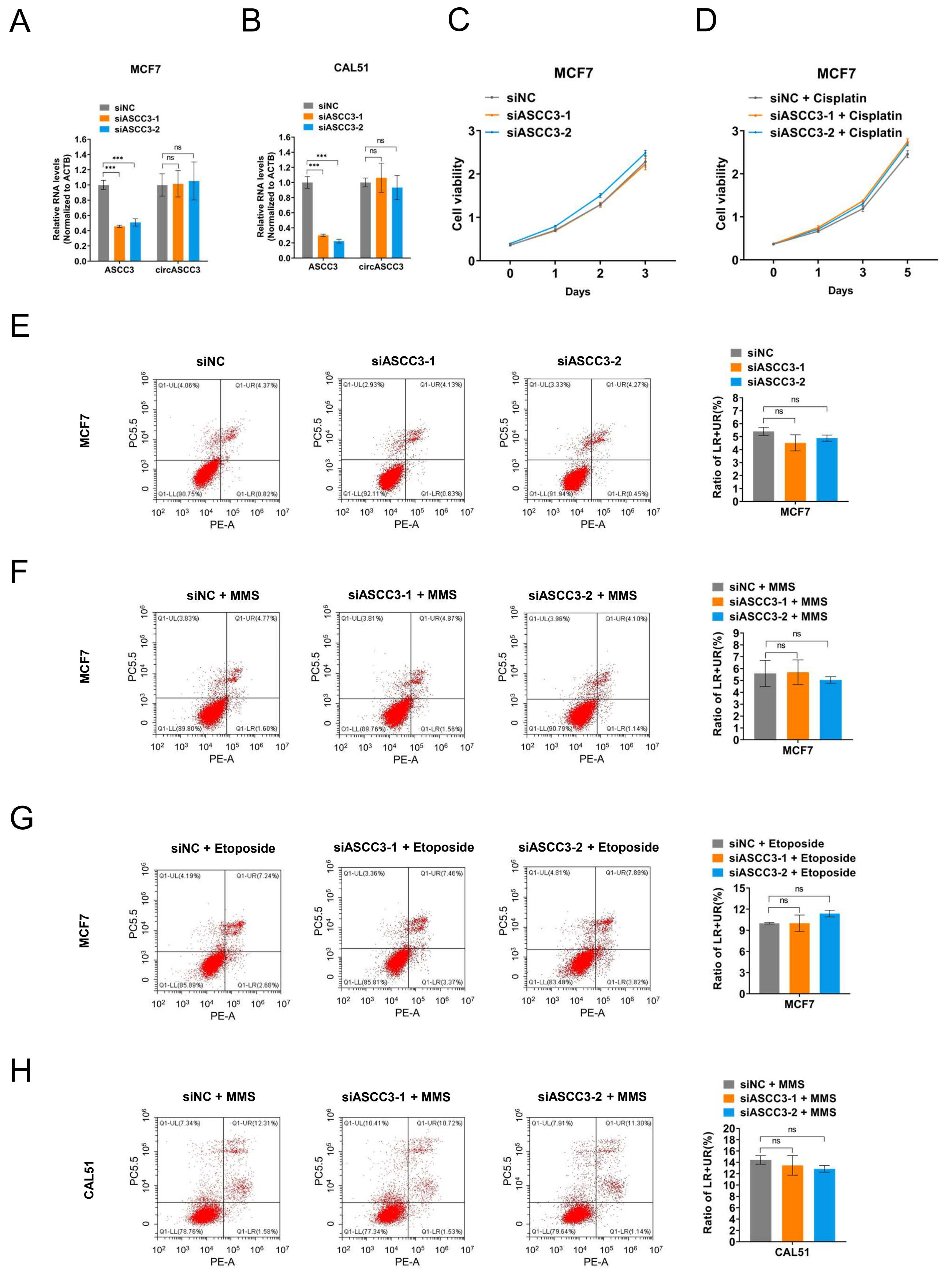
A
MCF7
siNC
siASCC3-1
siASCC3-2
Relative RNA levels (Normalized to ACTB)
ASCC3
circASCC3
B
CAL51
C
MCF7
Cell viability
Days
D
siNC + Cisplatin
siASCC3-1 + Cisplatin
siASCC3-2 + Cisplatin
E
F
siNC + MMS
siASCC3-1 + MMS
siASCC3-2 + MMS
G
siNC + Etoposide
siASCC3-1 + Etoposide
siASCC3-2 + Etoposide
H
CAL51
PC5.5
PE-A
Ratio of LR+UR(%)
ns

Figure S8

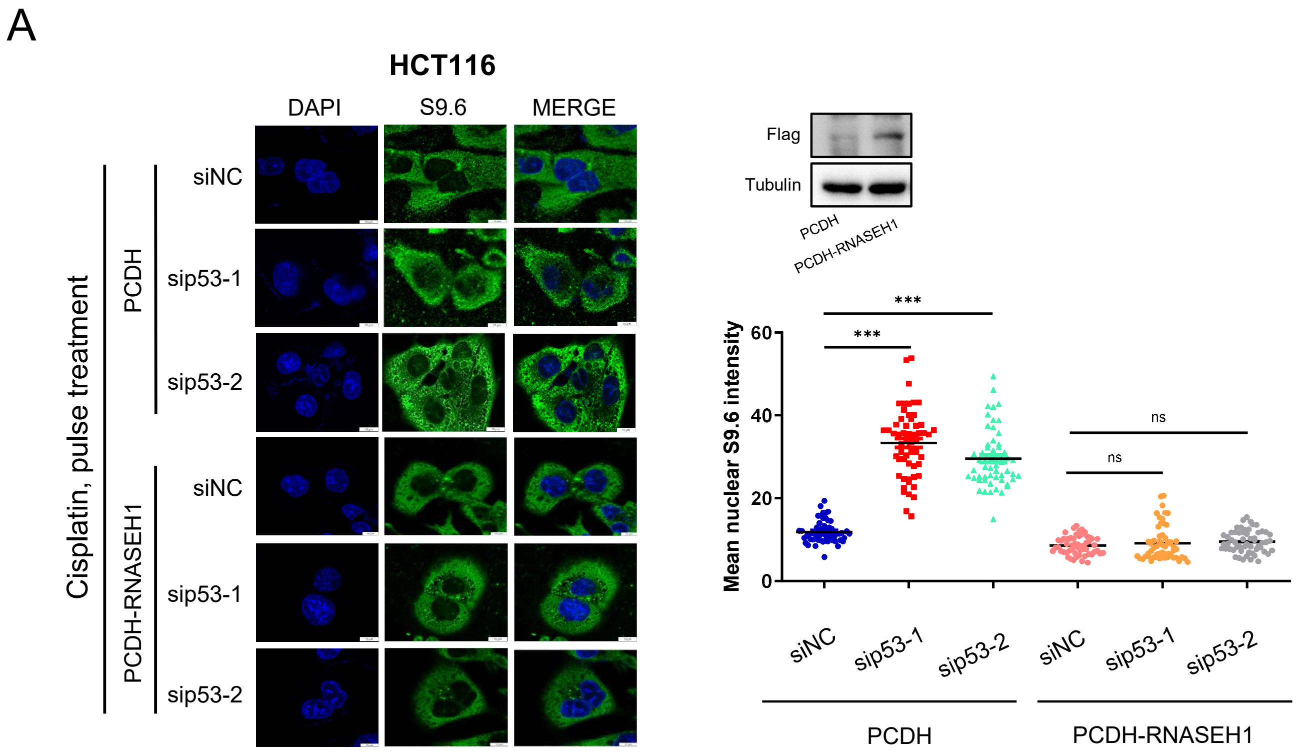
A
HCT116
DAPI
S9.6
MERGE
Cisplatin, pulse treatment
PCDH
PCDH-RNASEH1
siNC
sip53-1
sip53-2
Flag
Tubulin
PCDH
PCDH-RNASEH1
Mean nuclear S9.6 intensity
***
***
ns
ns
PCDH
PCDH-RNASEH1

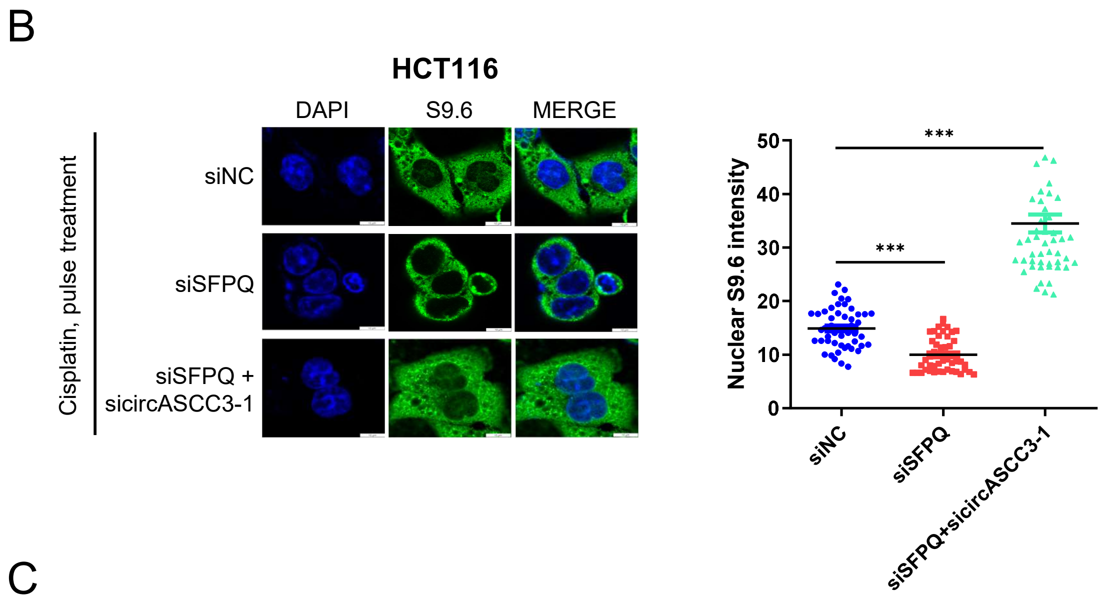
B
HCT116
DAPI
S9.6
MERGE
Cisplatin, pulse treatment
siNC
siSFPQ
siSFPQ + sicircASCC3-1
Nuclear S9.6 intensity
***
***
siNC
siSFPQ
siSFPQ+sicircASCC3-1

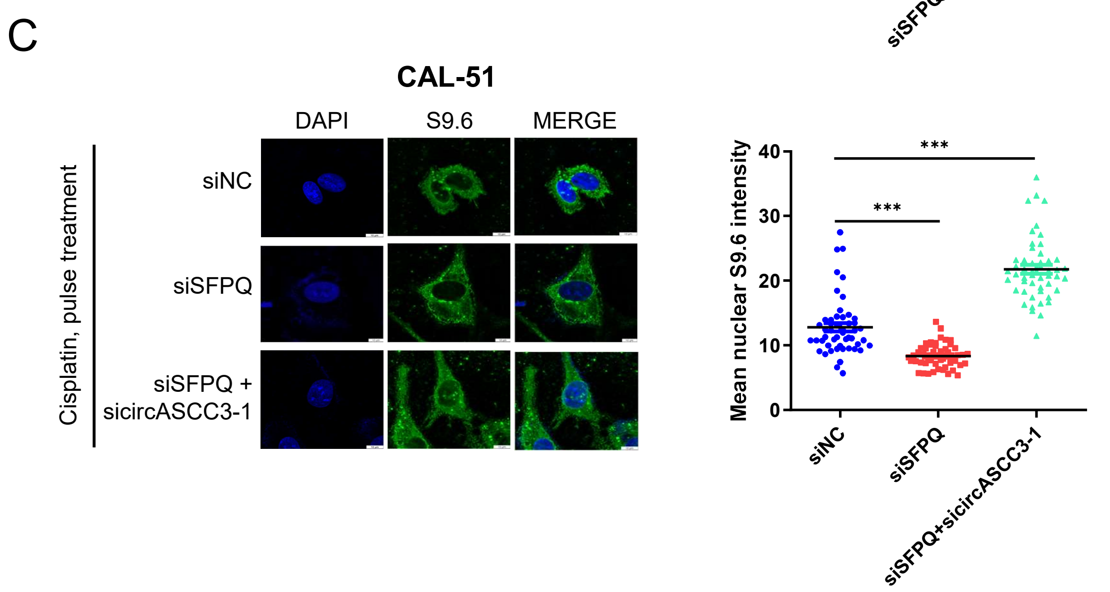
C
CAL-51
DAPI
S9.6
MERGE
Cisplatin, pulse treatment
siNC
siSFPQ
siSFPQ + sicircASCC3-1
Mean nuclear S9.6 intensity
***
***
siNC
siSFPQ
siSFPQ+sicircASCC3-1

Figure S9

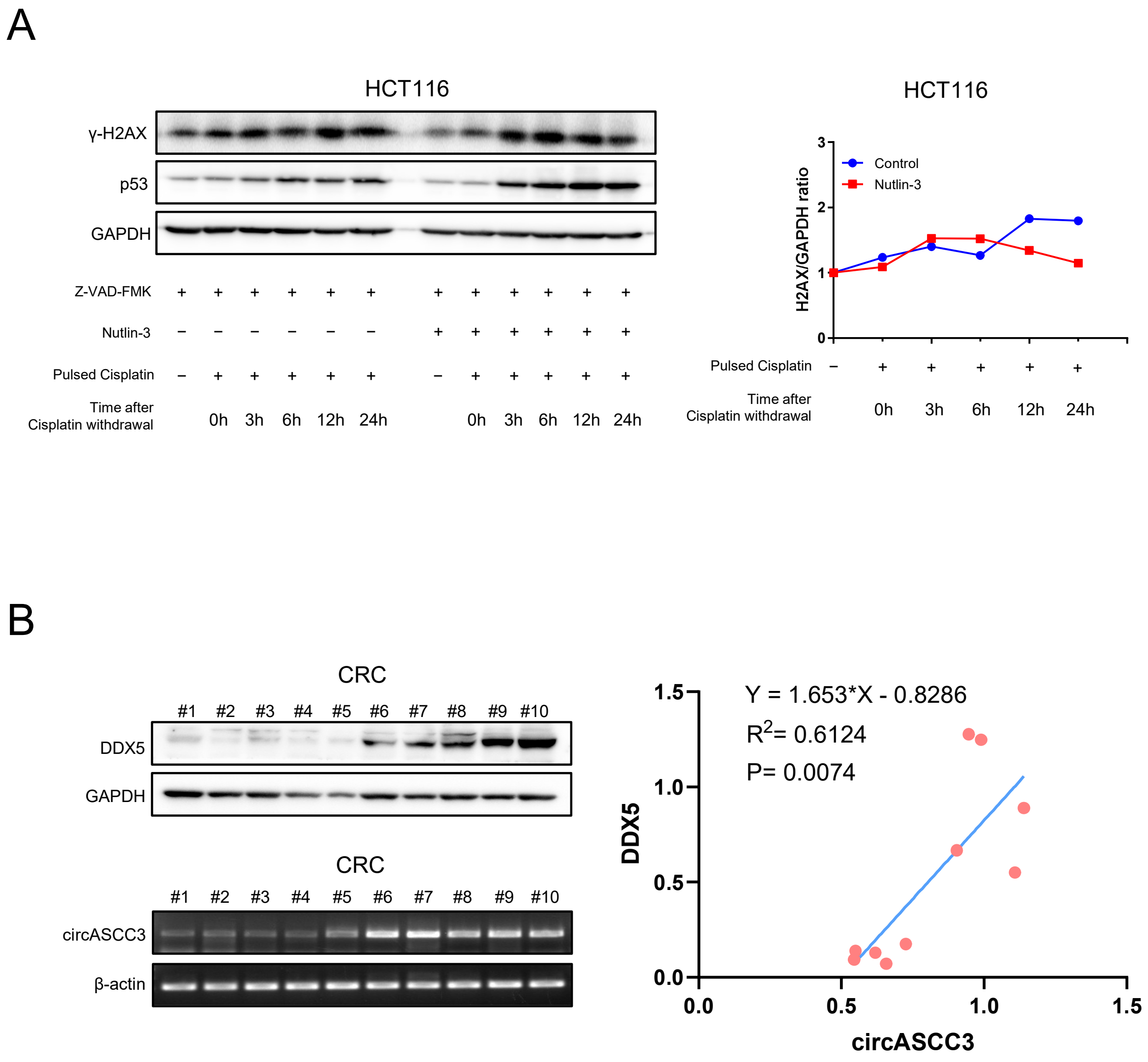
A
HCT116
γ-H2AX
p53
GAPDH
Z-VAD-FMK
Nutlin-3
Pulsed Cisplatin
Time after Cisplatin withdrawal
0h 3h 6h 12h 24h 0h 3h 6h 12h 24h
HCT116
H2AX/GAPDH ratio
Control
Nutlin-3
Pulsed Cisplatin
Time after Cisplatin withdrawal
0h 3h 6h 12h 24h
B
CRC
#1 #2 #3 #4 #5 #6 #7 #8 #9 #10
DDX5
GAPDH
CRC
#1 #2 #3 #4 #5 #6 #7 #8 #9 #10
circASCC3
β-actin
Y = 1.653*X - 0.8286
R2= 0.6124
P= 0.0074
DDX5
circASCC3

Supplementary Tables

Supplementary Table S1. Correlation between circASCC3 expression and clinicopathological characteristics

	variables	circASCC3 expression		total	χ^2	p value
		low	high			
Age (year)					6.050	0.014
	≤65	36	4	40		
	>65	27	13	40		
Sex					2.797	0.094
	Female	29	4	33		
	male	34	13	47		
Grade					0.025	0.875
	I/II	47	13	60		
	III	16	4	20		
TNM					0.043	0.835
	I/II	32	9	41		
	III/IV	28	7	35		
T					0.863	0.353
	II/III	18	3	21		
	IV	41	13	54		
N					0.043	0.835
	N0	32	9	41		
	N1/N2	28	7	35		
					0.273	0.601
M	M0	62	17	79		
	M1	1	0	1		

Supplementary Table S2. Univariate and multivariate analyses of the factors correlated with overall survival of colorectal cancer patients

variables	Univariate analysis				Multivariate analysis			
	p value	HR	95%CI		p value	HR	95%CI	
circASCC3 expression	0.129	1.675	(0.861	3.259)				
Sex	0.949	1.02	(0.559	1.861)				
Age	0.042	1.868	(1.021	3.414)	0.181	1.571	(0.811	3.046)
Grade stage	0.259	1.453	(0.76	2.779)				
TNM	0.006	2.443	(1.3	4.59)	0.066	1.881	(0.959	3.691)
T	0.015	2.948	(1.238	7.024)	0.074	2.275	(0.924	5.602)
N	0.006	2.443	(1.3	4.59)	0.066	1.881	(0.959	3.691)
M	0.415	2.294	(0.312	16.866)				

Supplementary Table S3. Primers for plasmid construction

Primer	Sequence (5'-3')
DDX5-Nhel-Myc-his-pcDNA3.1-F	CTAGCTAGCATGTCGGGTTATTCGAG
DDX5-Xhol-Myc-his-pcDNA3.1-R	CCGCTCGAGATTGGGAATATCCTGTT
NONO-Nhel-Myc-his-pcDNA3.1-F	GCGCTAGCTAGCATGCAGAGTAATAAAACTT
NONO-Xhol-Myc-his-pcDNA3.1-R	CCGCTCGAGTGTATCGGCGACGTTTGTT
RUVBL1-Nhel-Myc-his-pcDNA3.1-F	CTAGCTAGCATGAAGATTGAGGAGGTGAAGAGCACT
RUVBL1-Xhol-Myc-his-pcDNA3.1-R	CCGCTCGAGTCTTCATGTACTTATCCTGCTGGTC
EIF4A3-Nhel-Myc-his-pcDNA3.1-F	ATACTAGCTAGCATGGCGACCACGGCCACGA
EIF4A3-Xhol-Myc-his-pcDNA3.1-R	CCGCTCGAGCGATAAGATCAGCAACGTT
circASCC3-EcoRI-pLCDH-F	CGGAATTCTGAAATATGCTATCTTACAGCTATTTGAACTGCTGGGACCTGA
circASCC3-BamHI-pLCDH-R	CGGGATCCTCAAGAAAAAATATATTCACCTCATCTAAGTCTTGGATATAAACTGGC
ASCC3-KpnI-pGL3.0-F	CGGGGTACCCAAGTCCTTACACACAATGGTATA
ASCC3-NheI-pGL3.0-R	CTAGCTAGCAACGTCGGCACGTCGACTCGT
RNASEH1-EcoRI-Flag-PCDH-F	GATGACGATGACAAGGAATTCATGAGCTGGCTTCTGTTCCTGG
RNASEH1-BamHI -Flag-PCDH-R	CAGCTCGAGCCCGGGGGATCCTCAGTCTTCCGATTGTTTAGCTCC

Supplementary Table S4. shRNA sequences

Primer	Sequence (5'-3')
shNC-F	CCGGTTCTCCGAACGTGTCACGTTTCAAGAGAACGTGACACGTTCGGAGAATTTTTG
shNC-R	AATTCAAAAAATTCTCCGAACGTGTCACGTTCTCTTGAAACGTGACACGTTCGGAGAA
shcircASCC3-1-F	CCGGAAGACTTAGATGAGCTATT CTCGAGAATAGCTCATCTAAGTCTTTTTTTG
shcircASCC3-1-R	AATTCAAAAAAAGACTTAGATGAGCTATTCTCGAGAATAGCTCATCTAAGTCTT
shcircASCC3-2-F	CCGGCTTAGATGAGCTATTTGAATTCAAGAGATTCAAATAGCTCATCTAAGTTTTTG
shcircASCC3-2-R	AATTCAAAAACTTAGATGAGCTATTTGAATCTCTTGAATTCAAATAGCTCATCTAAG

Supplementary Table S5. siRNA sequences

Primer	Sequence (5’-3’)
siNC	TTCTCCGAACGTGTCACGT
sicircASCC3-1	AAGACTTAGATGAGCTATT
sicircASCC3-2	CTTAGATGAGCTATTTGAA
siASCC3-1	GCGATCTAAACTTCATGAA
siASCC3-2	GCTCGAAATGCCACTGTAA
siDDX5	GCAATACGGAAGTAATGTT
sip53-1	GTAATCTACTGGGACGGAA
sip53-2	AACTACATGTGTAACAGTTCCT
siSFPQ-1	GTACGAATATTCTCAGCGA
siSFPQ-2	GGAAGATGCCTATCATGAA
siCPEB4	GGATCGAATCTCCAGTGTT
siFUBP3	CATCAAAGGACGGTAATAA
siHNRNPA1	CCACGAAACCAAGGTGGCT
siHNRNPL	CGGATGTTCTTTACACTAT
siHNRNPM	CCTGAATAAAGGCATCGGA
siHNRNPU	GGTAGCTGAGTGCTTTGAT
siKHDRBS1	CCACAAGGGAATACAATCA
siKHSRP	GGACGGATCTCAGAATACG
siMATR3	GGAGCTCATAGTGCACTGT
siPABPC4	CGGCAGGCAGAGTTAAAAC
siPUM2	GCATGGTAGAATATGTATT
siTARDBP	GGATGAACTTTGGTGCGTT

Supplementary Table S6. Primers for RT-qPCR

Primer	Sequence (5'-3')
SFPQ-F	CCTCCATCGAGTCCCTTCCT
SFPQ-R	AACTAAGTCATCCCACGAGCC
p53-F	GCCATGGAGGAGCCGCAGTCAGAT
p53-R	TCAGTCTGAGTCAGGCCCTTCTGT
DDX5-F	TCGCAGTACCAAAACAGGCA
DDX5-R	AGTATCTGTCCCGACGGTCA
CPEB4-F	ACATCTAGCGCATCGTCTCTT
CPEB4-R	ACAACAGAGCACCGTTATTAGC
FUBP3-F	TCCGGCAGATTGCTGCTAAAA
FUBP3-R	CCGTATCCATATACTGAGGGGTC
HNRNPA1-F	TCAGAGTCTCCTAAAGAGCCC
HNRNPA1-R	ACCTTGTGTGGCCTTGCAT
HNRNPL-F	TACGCAGCCGACAACCAAATA
HNRNPL-R	CTCCGGGAGTCATCCGAGT
HNRNPM-F	GCGGCGACGGAGATCAAAA
HNRNPM-R	CTCATTCTGAGCAGGTCGTTC
HNRNPU-F	GGGGACGGCAAAACAGAACA
HNRNPU-R	AGCACTGAGACGATCTCTTGA
KHDRBS1-F	GGAGCCAGAGAACAAGTACCT
KHDRBS1-R	CATGGCGTGAGTGAAGGAC
KHSRP-F	ATCCGCAAGGACGCTTTCG
KHSRP-R	TGCTCTCCGGTTGATCTCCAT
MATR3-F	ATCAATGGAGCAAGTCACAGTC
MATR3-R	TGCAACATGAATGGATCACCC
PABPC4-F	AAGCCAATCCGCATCATGTG
PABPC4-R	CTCTTGGGTCTCGAAGTGGAC
PUM2-F	TCGGGGAATGGGAGAGCTTT
PUM2-R	GCTGGGACATTGAATGGTGAGA
TARDBP-F	GGGTAACCGAAGATGAGAACG
TARDBP-R	CTGGGCTGTAACCGTGGAG

Supplementary Table S7. Primers for circASCC3 identification and RNA stability analysis

Primer	Sequence (5'-3')
circASCC3 convergent-F	TTTTAGGAGAAAATGCTAAACCCAA
circASCC3 convergent-R	CCGCAATTCCTTAGGATCAAAGC
circASCC3 divergent-F	GGATTCCCTACAGCGAACCAA
circASCC3 divergent-R	CTTGAAGAGCCTGAAACCTATGA
circNSUN2 convergent-F	GTATCCTGAAGAACTTGCCTGG
circNSUN2 convergent-R	GAAACTGATGAAACTTTTCCAAGTG
circNSUN2 divergent-F	CCACCACTGCTCCTCAAC
circNSUN2 divergent-R	AAGTGTGGCGATTTTCTCAAG
ACTB convergent-F	AACCTAACTTGCGCAGAAAACA
ACTB convergent-R	CTGTAACAACGCATCTCATATTTGG
ACTB divergent-F	CTGTACGCCAACACAGTGC
ACTB divergent-R	GTACTTCAGGGTGAGGATGCC
18S-F	AAACGGCTACCACATCCAAG
18S-R	CCTCCAATGGATCCTCGTTA
circASCC3-F	GTTCCAATTCTGAGCAGGCAG
circASCC3-R	TTCAAGTCCTTCAGGTCCCA
ASCC3-F	CCTCCATCGAGTCCCTTCCT
ASCC3-R	AACTAAGTCATCCCACGAGCC
ACTB-F	CAAGAGAGGCATCCTCACCCT
ACTB-R	GGATAGCACAGCCTGGATAGCAA
U1-F	TCCCAGGGCGAGGCTTATCCATT
U1-R	GAACGCAGTCCCCCACTACCACAAAT
circNSUN2-F	CCACCACTGCTCCTCAAC
circNSUN2-R	AAGTGTGGCGATTTTCTCAAG
p21-F	CTGGACTGTTTTCTCTCGGCTC
p21-R	TGTATATTCAGCATTGTGGGAGGA

Supplementary Table S8. Primers for in vitro transcription in RNA-pull down

Primer	Sequence (5'-3')
T7-Linear sense I-F	TAATACGACTCACTATAGGGAGACTATTTGAACTGCTGGGACC
Linear sense I-R	CTCATCTAAGTCTTGGATATAAACTGGC
T7-Linear antisense I-F	TAATACGACTCACTATAGGGAGACTCATCTAAGTCTTGGATATAAAC
Linear antisense I-R	CTATTTGAACTGCTGGGACCTGAAGGAC
T7-Linear sense II-F	TAATACGACTCACTATAGGGAGAATGATTTTGCCAGAAGGAATCCA
Linear sense II-R	CTTTGCACCAGCAATAAATGCTGATGT
T7-Linear antisense II-F	TAATACGACTCACTATAGGGAGACTTTGCACCAGCAATAAATGCT
Linear antisense II-R	ATGATTTTGCCAGAAGGAATCCAAAGA
T7-CTRL probe-F	TAATACGACTCACTATAGGGAGAGCCAGTTTATATCC
CTRL probe-R	CAGGTCCCAGCAGTTCAAATAGCTCATC
T7-circASCC3 probe-F	TAATACGACTCACTATAGGGAGACAGGTCCCAGC
circASCC3 probe-R	GCCAGTTTATATCCAAGACTTAGATGAGCT

Supplementary Table S9. Primers for RIP-qPCR

Primer	Sequence (5’-3’)
BS-1-F	GAGCATGCCTTCAGAGTAGCA
BS-1-R	CCCATTATGTCATATGCCCTGG
BS-2-F	AAATGCCTATCATAGTACCTGGCA
BS-2-R	GCTGTTCATTTTACTGAAGTTATGC
BS-3-F	GTTTGCCTGTGGAAAATTGGCA
BS-3-R	TGCTGTTTTTGCTTGCCTGTATTAT
BS-4-F	TCACTGTAGATATTGATCCTGCCA
BS-4-R	TCTCAGTGAAAAACGACAGACCT
BS-5-F	GAAGGTCTAGAGTAGAGCTGCTG
BS-5-R	TTCAGGGGTCTTGCTCCTAA
BS-6-F	AGAGAAGCATAAAAGTCAACGGG
BS-6-R	CTACACTGTACCCCCAAAAGGG

Supplementary Table S10. Primers for ChIP-qPCR

Primer	Sequence (5’-3’)
(ASCC3) p53-RE-1-F	GCACCTCTCATTATCACTTGGC
(ASCC3) p53-RE-1-R	GTGTCTTTGGTCCTGGACTTCAT
(ASCC3) p53-RE-2-F	GTAATCGACATAAGAAT
(ASCC3) p53-RE-2-R	GGGGTAGCCACCAGTTAAATACGT
(ASCC3) p53-RE-3-F	TCCCTTAAAAGGACATCAAGTTGG
(ASCC3) p53-RE-3-R	TACAAGGTGGTGACCAAACACA
(SFPQ) p53-RE-1-F	TGTGTCATCCGCCATTTTGTGAG
(SFPQ) p53-RE-1-R	GAGAACGGAAGTCGTGGAGG
(SFPQ) p53-RE-2-F	GATTACAGGCGTCAGCCACC
(SFPQ) p53-RE-2-R	CAATGTTAGGCTGGGCACTGT
(SFPQ) p53-RE-3-F	ATTACAGGTGCATGCCACCAC
(SFPQ) p53-RE-3-R	AGGTCCACAGACCTTTTGCG

Supplementary Table S11. Primers for DRIP and R-ChIP

Primer	Sequence (5’-3’)
ACTB Pause-F	GGGACTATTTGGGGGTGTCT
ACTB Pause-R	TCCCATAGGTGAAGGCAAAG
RPS23-F	TTAGTCGGTTCAGGGCAACTTGA
RPS23-R	CTAAGACACTCGCCTCACCTGGA
JUN-TSS-F	GGGTGACATCATGGGCTATT
JUN-TSS-R	TCGGACTATACTGCCGACCT
NEAT1-TSS-F	TAGTTGTGGGGGAGGAAGTG
NEAT1-TSS-R	ACCCTGCGGATATTTTCCAT
